# The homeoprotein HOXB2 limits triple-negative breast carcinogenesis via extracellular matrix remodeling

**DOI:** 10.7150/ijbs.88837

**Published:** 2024-01-20

**Authors:** Ji Hoon Oh, Clara Yuri Kim, Da Som Jeong, Yu Cheon Kim, Myoung Hee Kim, Je-Yoel Cho

**Affiliations:** 1 Department of Biological Sciences, Keimyung University College of Natural Sciences, Daegu, Republic of Korea.; 2 Department of Anatomy, Embryology Laboratory, Yonsei University College of Medicine, Seoul, Republic of Korea.; 3 Department of Anatomy, Graduate School of Medical Science, Brain Korea 21 Project, Yonsei University College of Medicine, Seoul, Republic of Korea.; 4 Department of Biochemistry, Brain Korea 21 Project and Research Institute for Veterinary Science, Seoul National University College of Veterinary Medicine, Seoul, Republic of Korea.; 5 Comparative Medicine Disease Research Center, Seoul National University, Seoul, Republic of Korea.

**Keywords:** HOXB2, triple-negative breast cancer, cancer metastasis, ECM, EMT

## Abstract

Homeobox genes and their encoded DNA-binding homeoproteins are master regulators of development. Consequently, these homeotic elements may regulate key steps in cancer pathogenesis. Here, using a combination of *in silico* analyses of large-scale patient datasets, *in vitro* RNAi phenotyping, and *in vivo* validation studies, we investigated the role of HOXB2 in different molecular subtypes of human breast cancer (BC). The gene expression signatures of HOXB2 are different across distinct BC subtypes due to various genetic alterations, but HOXB2 was specifically downregulated in the aggressive triple-negative subtype (TNBC). We found that the reduced expression of HOXB2 was correlated with the metastatic abilities (epithelial-to-mesenchymal transition) of TNBC cells. Further, we revealed that HOXB2 restrained TNBC aggressiveness by ECM organization. HOXB2 bound to the promoter regions of *MATN3* and *ECM2* and regulated their transcription levels. Forced expression of HOXB2 effectively prevented TNBC progression and metastasis in a mouse xenograft model. Reduction of HOXB2 and the *HOXB2*/*MATN3*/*ECM2* transcriptional axis correlated with poor survival in patients with various cancers. Further, we found the long non-coding RNA HOXB-AS1 in complex with SMYD3, a lysine methyltransferase, as an epigenetic switch controlling HOXB2 expression. Overall, our results indicate a tumor-suppressive role of HOXB2 by maintaining ECM organization and delineate potential clinical utility of HOXB2 as a marker for TNBC patients.

## Introduction

Triple-negative breast cancer (TNBC) is the most aggressive and invasive breast cancer subtype^1^. TNBC does not express the estrogen receptor (ER) or the progesterone receptor (PR) and lacks the overexpression of human epidermal growth factor receptor 2 (HER2) as found in other breast cancers^1^. This deficiency in molecular targets results in poor prognosis and survival as no effective therapeutic agents have yet been identified, with surgery a common treatment strategy^2^. Thus, although TNBC only accounts for approximately 15% of breast cancer diagnoses, further research is crucial to identify practical, non-invasive treatment options.

*HOX* genes, a group of well-conserved genes that share a conserved homeobox, are involved in numerous processes and play pivotal roles in embryonic development^3^. Recently, various *HOX* genes have been revealed to function in cancer as oncogenes or tumor suppressors^4^. Among these *HOX* genes, HOXB2 is best known for contributing to tumorigenesis in multiple cancers such as breast, lung, cervical, and esophageal cancer^5^-^10^. Nevertheless, its potential functions and associated molecular pathways in cancer and the invasive phenotypes associated with cancer remain unknown.

Long non-coding RNAs (lncRNAs) are transcripts longer than 200 nucleotides but not translated^11^. The lncRNA HOXB-AS1 (HOXB cluster antisense RNA 1) adjacent to the HOXB2 gene has been reported to be involved in tumorigenesis and progression of several carcinomas^9^, ^12^. In glioblastoma, HOXB-AS1 promotes proliferation, migration, and invasion of glioblastoma cells through the HOXB-AS1/miR-885-3p/HOXB2 axis^9^. In endometrial carcinoma, HOXB-AS1 functions as a sponge by binding to miR-149-3p to increase Wnt10b and downstream target genes^12^. However, the involvement and mechanisms by which HOXB-AS1 regulates TNBC progression through HOXB2 are not well understood.

SET and MYND domain-containing proteins 3 (SMYD3), a member of the SMYD family (SMYD1, -2, -3, -4, and -5), is a lysine methyltransferase of histone protein^13^. As a chromatin modifier, SMYD3 tri-methylates on histone 3 lysine 4 (H3K4) and activates transcription by increasing chromatin accessibility at the gene promoter region^14^. SMYD3 plays an vital role in the initiation and progression of breast cancers by regulating cancer cell proliferation, migration, and invasion^15^, ^16^. A previous study demonstrated that SMYD3 promotes epithelial-to-mesenchymal transition (EMT) through TGF-β signaling in MDA-MB-231 cells^16^. Moreover, dysregulated expression of SMYD3 in TNBC patients has been observed^17^. However, the precise mechanisms underlying the function of SMYD3 in TNBC progression and poor prognosis remain unclear.

A typical therapeutic strategy is to reduce the migratory and invasive characteristics of cancer. In particular, the extracellular matrix (ECM) and its dynamics constitute important regulators of these features. The matrix consists of various proteins, such as collagens, laminins, and matricellular proteins, which help define its structure and function^18^, ^19^. Notably, ECM composition continuously changes during tumorigenesis to aid in cancer cell fitness and metastasis^20^, ^21^. For example, during EMT, ECM is degraded by matrix metalloproteinases, facilitating the transformation of migratory mesenchymal cells to invade other areas of the body readily^22^. Of the many EMT- and ECM-associated factors, matrilin-3 (MATN3), and extracellular matrix protein 2 (ECM2) have been related to cancer metastases. MATN3 is associated with gastric cancer recurrence and regulates the expression of ECM-degrading proteins such as MMP1, MMP3, and MMP13^23^. In addition, ECM2, also known as secreted protein acidic and rich in cysteine (SPARC)-like1 (SPARCL1), functions as an antagonist of cell adhesion. Overexpression of ECM2 suppresses cell migration in hilar cholangiocarcinoma by inhibiting MMP-9 and MMP-2^24^ and in trophoblast cells by regulating MMP-2 and MMP-3 expression^25^. However, whether dysregulation of these genes is involved in TNBC tumorigenesis has not yet been explored.

Here, we identified that HOXB2 is positively regulated by HOXB-AS1/SMYD3 complex. SMYD3 binding at HOXB2 promoter guided by HOXB-AS1 leads to histone H3 lysine 4 trimethylation (H3K4me3) enrichment, resulting in the active transcription of HOXB2. Additionally, we demonstrated that HOXB2 is downregulated in TNBC and plays a key role in TNBC invasiveness and metastasis by modulating ECM organization. HOXB2 depletion resulted in accelerated migratory and invasive phenotypes by regulating the expression of EMT markers such as CDH1, ITGB4, CDH2, and VIM. Using a mouse xenograft model, increasing HOXB2 expression effectively prevents TNBC progression and metastasis. Furthermore, we showed that HOXB2 is associated with a network of downstream molecules responsible for ECM formation in breast cancer cells. In particular, MATN3 and ECM2, identified as transcriptional targets of HOXB2 through promoter assays, were shown to promote aggressive TNBC characteristics. Together, our findings reveal that HOXB2 constitutes a novel therapeutic indicator for TNBC by regulating the ECM components, MATN3 and ECM2, and EMT, thus exhibiting potential clinical and therapeutic relevance.

## Materials and Methods

### Cell lines and culture conditions

Parental BT474, MCF7, T47D, MDA-MB-453, and MDA-MB-231 cell lines were provided by Drs. Yong Nyun Kim and Kyung Tae Kim (National Cancer Center, Korea). MDA-MB-157 and MDA-MB-468 cell lines were provided by Prof. Jong Hoon Park (Sookmyung women's University, Korea). These breast cancer cell lines were cultured as previously described^26^, and periodically tested for mycoplasma contamination. For overexpression studies, cells were transfected with the GFP-HOXB2 plasmid (pCMV6-AC-GFP vector backbone; Origene, Rockville, MD, USA) for 24 h using the Attractene Transfection Reagent (Qiagen, Hilden, Germany) according to the manufacturer's protocol. GFP-HOXB2 plasmid-transfected cells were treated with neomycin (0.5 mg/ml; Thermo Fisher Scientific, Waltham, MA, USA) for 2 weeks to generate HOXB2-overexpressing stable cell lines. For transient knockdown studies, HOXB2-siRNA (Genolution, Seoul, Korea), HOXB-AS1-siRNA (Genolution), MATN3-siRNA (Genolution), SMOC2-siRNA (Genolution), ECM2-siRNA (Genolution), COL8A2-siRNA (Genolution), and negative control siRNA were transfected at a final concentration of 40 nM for 48 h. Sequences of siRNAs are available in Table S1.

### Real-time quantitative polymerase chain reaction (qPCR)

Real-time qPCR analysis was performed using the StepOnePlus™ Real-Time PCR System (Applied Biosystems, Foster City, CA, USA) and Power SYBR Green PCR Master Mix (Applied Biosystems) kits. All samples were analyzed in triplicate and *HOXB2* expression was normalized relative to that of *β-Actin*, which was used as an internal loading control. Primers for PCR were listed in Table S2.

### Western blotting and antibodies

For western blot analysis, each cell line was treated under appropriate conditions and lysed. After determining the protein concentration, equal amount of cell lysate was loaded onto 8% SDS-PAGE gel and blotted. Anti-HOXB2 (1:1000, ab220390, Abcam, Cambridge, UK), anti-E-cadherin (1:10000, ab40772, Abcam), anti-integrin β4 (1:2000, ab133682, Abcam), anti-N-cadherin (1:1000, ab18203, Abcam), anti-vimentin (1:5000, ab92547, Abcam), anti-GAPDH (1:10000, ab181602, Abcam), and anti-β-Actin (1:10000, ab6276, Abcam) antibodies were used to detect each protein.

### Transwell invasion assay

Transwell invasion assay was performed using Matrigel™ (BD Biosciences, Franklin Lakes, NJ, USA). The migration assay was performed using the same protocol in the absence of Matrigel. For the experiments, 5 × 10^4^ cells were placed in each chamber. After a cell line-dependent 24-72 h incubation, invading cells were stained with fluorochrome 4,6-diamidino-2-phenylindole (DAPI) and observed via fluorescence microscopy (Nikon Instruments Inc., Melville, NY, USA). The acquired images were analyzed using ImageJ software (National Institutes of Health, Bethesda, MD, USA).

### Migration assay

Cells were cultured in 24-well culture vessels at a density of 6 × 10^5^ cells/well until confluent, then straight scratches were made in the monolayers using 200-µl pipette tips. After washing with culture medium to remove cell debris, the scratched monolayers were incubated in culture medium, the scratched gaps were photographed at regular intervals (0, 12, and 24 h), and the cell-free scratched areas were measured. Results were obtained from at least three independent experiments.

### Immunocytochemical (ICC) analysis

ICC analyses were performed using the manufacturer's ICC protocol (Abcam). Anti-E-cadherin (1:300, ab40772, Abcam), anti-integrin β4 (1:300, ab133682, Abcam), anti-N-cadherin (1:500, ab18203, Abcam), and anti-vimentin (1:500, ab92547, Abcam) were used to detect each protein. Nuclei were counterstained with DAPI. Images were captured with a 40 × C-Apochromat water immersion lens on a Zeiss LSM 700 confocal microscope (Oberkochen, Germany), using Zen 2011 software.

### Chromatin immunoprecipitation (ChIP) assay

ChIP analysis was performed as previously described with minor modifications^27^. Anti-HOXB2 (ab220390, Abcam), anti-H3K4me3 (ab1012, Abcam), anti H3K27me3 (ab6002, Abcam), anti-H3K9ac (ab12179, Abcam), anti-SMYD3 (ab228015, Abcam) and non-immune mouse IgG (sc2025, Santa Cruz, CA, USA) were used for immunoprecipitation. ChIP-PCR data are shown as the percentage of input following normalization with IgG. The primers used for ChIP-PCR were listed in Table S2.

### RNA immunoprecipitation (RIP) assay

For RIP assay, 1 × 10^7^ cells/antibody were collected and lysed on ice in RIP buffer containing protease inhibitors for 10 m, then sonicated with a Sonics Vibra Cell^TM^ (5 m: pulse 10 s, interval 10 s) on ice. The lysates were centrifuged at 12,000 × g at 4 °C for 5 m and the supernatant was taken. The samples were first incubated with anti-SMYD3 (ab228015, Abcam), anti-RNA Pol II (ab817, Abcam), anti-SP1 (ab13370, Abcam), and non-immune mouse IgG (sc2025, Santa Cruz) antibodies at 4 °C for at least 4 h, and then protein-coated A/G agarose beads (Santa Cruz) were added and incubated overnight at 4 °C with gentle shaking. On the following day, the immunoprecipitated eluates were resuspended in 1 ml TRIzol (Invitrogen^TM^, Carlsbad, CA, USA), and RNA was extracted. The desired product was detected by real-time qPCR.

### Dual luciferase assay

For dual luciferase assay, genomic DNA fragments of the *MATN3* and *ECM2* promoter regions were cloned into the pGL3-Basic vector (Promega, Madison, WI, USA). The control pGL3-Basic vector, pGL3-MATN3, or pGL3-ECM2 constructs were transfected into HEK293T cells along with the *Renilla* luciferase vector (pRL Renilla luciferase control).

### Tissue microarray and immunohistochemical (TMA-IHC) analyses

The formalin-fixed paraffin-embedded human breast carcinoma tissue microarray containing 100 cases of invasive carcinoma of no special type was obtained from US Biomax (BC081116d, US Biomax Inc., Rockville, MD, USA). Before deparaffinization with xylene, slides were baked in an incubator at 60 °C for 1 h. Rehydration was performed with gradually reduced concentrations of ethanol from 100% to 95%, 85%, and 70% for 1 m. For antigen retrieval, the slides were submerged in citrate buffer and boiled in a microwave for 10 m. Sufficiently cooled slides were stained using Novolink Polymer Detection Systems (Leica Biosystems Inc., Buffalo Grove, IL, USA) according to the manufacturer's instructions. The anti-HOXB2 (1:200, ab220390, Abcam), anti-MATN3 (1:100, ab238893, Abcam), and anti-ECM2 (1:300, ab122268, Abcam) antibodies were diluted with phosphate buffered saline with Tween 20 (PBST) buffer. DAB staining was carried out until the tissue sections expressed a brown color. Tissue samples were then dehydrated with 70%, 85%, 95%, and 100% ethanol and cleared with xylene. The DAB signals of 100 breast carcinomas were scored using ImageJ software. Negative/low and high levels were classified based on the score of each protein signal intensity and area.

### Animal experiments

All animal experiments were approved by the Institutional Animal Care and Use Committee of Yonsei University (IACUC number: 2020-0197) and were conducted in compliance with the Guide for the Care and Use of Laboratory Animals. BALB/c nude mice were purchased from Orient Bio Inc. (Seongnam, Gyeonggi-do, Korea). For *in vivo* xenograft experiments, 5 mice per cell group were used. All mice were maintained under 12 h light/12 h dark conditions in a specific pathogen-free barrier facility. For xenograft experiments, 2.0 × 10^6^ empty vector-overexpressing MDA-MB-231 (MDA-MB-231:Emp.Vec) cells and HOXB2-overexpressing MDA-MB-231 (MDA-MB-231:HOXB2) cells were suspended in a mixture of 100 µl PBS and Matrigel (BD Bioscience) (50:50 v/v) and subcutaneously injected into the fat pads or intravenously injected into the tail veins of the test animals. Tumor volume was monitored using a caliper for 40 days. When the tumor size reached 10 mm^3^, the volume of the tumor (V) was calculated using the following equation: V (mm^3^) = 3.14 × width (W) × length (L) × height (H)/6 of the tumor. After 8 weeks of injection, the mice were euthanized and visceral organs were dissected for analysis. In addition, tumor metastasis was evaluated at different time points and compared between mice injected with MDA-MB-231:Emp.Vec, and MDA-MB-231:HOXB2 cells. Metastasis was monitored using an *in vivo* imaging system (IVIS; Caliper Life Science, Hopkinton, MA, USA) for 5 days. At different time points of injection (12, 24, 48, 72, 96, and 120 h), the mice were euthanized and visceral organs were dissected for metastasis analysis.

### Bioinformatic analysis

Survival analysis was performed using the Kaplan-Meier plotter (http://kmplot.com) and Molecular Therapeutics for Cancer, Ireland (MTCI; http://glados.ucd.ie/BreastMark/index.html). The web-accessible database cBioPortal (https://www.cbioportal.org) was used to determine the genetic alteration of *HOXB2* in various breast cancer subtypes (TCGA, Nature 2012; METABRIC, Nature 2012 & Nat Commun 2016; and SMC 2018) and to confirm the expression level of *HOXB2* in metastatic breast cancer (Provisional, February 2020). The web-accessible database TANRIC (https://ibl.mdanderson.org/tanric/_design/basic/query.html) and CCLE (https://sites.broadinstitute.org/ccle) were used to any positive correlation between *HOXB2* and *HOXB-AS1*/*SMYD3* in patients with breast cancer. The GEO datasets (GSE65194 and GSE58812) were used to evaluate HOXB2 expression and any positive correlation between *HOXB2* and *MATN3*/*ECM2* in patients with breast cancer. DAVID v6.8 (https://david.ncifcrf.gov/home.jsp) was used for gene ontology (GO) analysis.

### Statistical analysis

Data are expressed as mean values with standard error of the mean. Statistical differences were determined using Student's *t*-test, one-way ANOVA, or Chi-squared test. A *p*-value of < 0.05 was considered to indicate statistical significance.

## Results

### Downregulation of HOXB2 in TNBC

**Figure 1:**
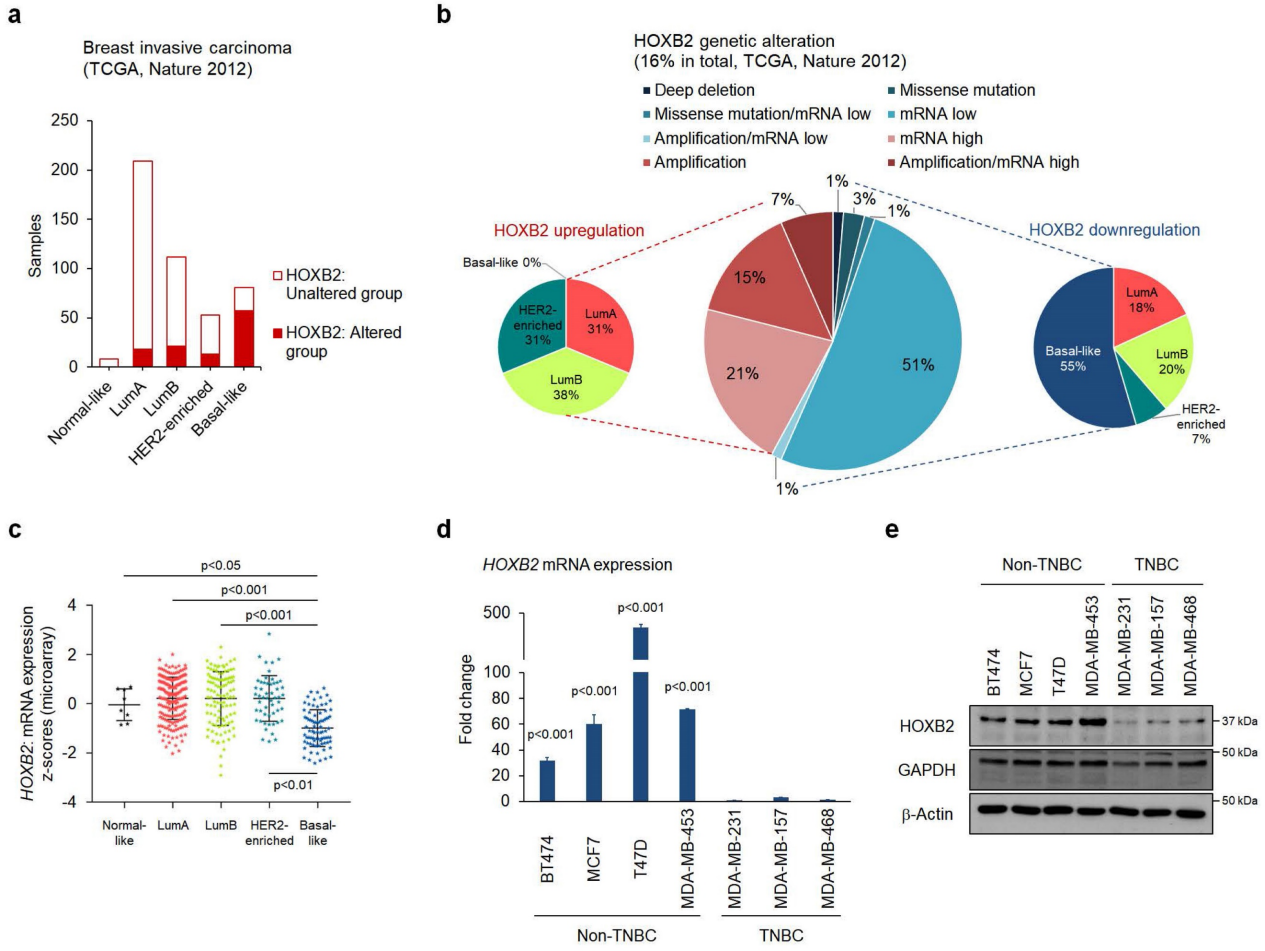
HOXB2 downregulation correlates with TNBC. **a** Public data retrieved from the cBioPortal displaying the distribution of breast cancer subtypes according to HOXB2 alteration. The Cancer Genome Atlas (TCGA, Nature 2012) dataset was used for this analysis. **b** Various HOXB2 abnormalities can be observed in breast cancer tissue samples. **c** Analyses of TCGA dataset (Nature 2012) for the expression of *HOXB2* in different breast cancer subtypes. **d** Detection of *HOXB2* mRNA expression in different human breast cancer cell lines by RT-qPCR. The fold change was calculated in respect to the expression of MDA-MB-231, a TNBC cell line with the lowest expression of *HOXB2*. Also, *β-Actin* was used to normalize to the RT-qPCR data. **e** Lysates from different human breast cancer cell lines were subjected to western blotting to examine HOXB2 protein levels.

To determine whether HOXB2 expression is altered among different breast cancer subtypes, the TCGA dataset was analyzed. The proportion of cases with HOXB2 alteration was significantly augmented in basal-like breast cancer compared to that in other subtypes (**Fig. 1a**). In particular, genetic alterations such as mRNA low and missense mutations, which ultimately result in HOXB2 downregulation, were especially enriched in the basal-like subtype, whereas genetic alterations responsible for HOXB2 upregulation were only found in luminal A, B, and HER2-enriched subtypes (**Fig. 1b**). To gain further insight into HOXB2 expression in breast cancer, we analyzed a microarray of gene expression from a publicly available TCGA dataset. The expression levels of *HOXB2* were similar between normal-like, luminal A, B, and HER2-enriched breast cancers; however, *HOXB2* expression was significantly lower in basal-like breast cancer (**Fig. 1c**). A comparable expression pattern was observed in a different dataset from GSE65194 (41 TNBC, 30 HER2-enriched breast cancer, and 29 luminal A and 30 luminal B breast cancer; Fig. S1a), METABRIC (199 TNBC; 220 HER2-enriched breast cancer; and 279 luminal A and 461 luminal B breast cancer; Fig. S1b), and SMC (36 TNBC; 18 HER2-enriched breast cancer; and 47 luminal A and 65 luminal B breast cancer datasets; Fig. S1c). Moreover, we examined the gene expression of the anterior *HOXB* genes (*HOXB1*, *B3*, and *B4*) adjacent to *HOXB2*, as *HOX* genes are regulated as clusters. Among the anterior *HOXB* genes, *HOXB3* was also downregulated in TNBC (Fig. S1d**-**f).

Therefore, we further investigated the expression of *HOXB2* and *HOXB3* and their correlation with the clinical characteristics of patients with breast cancer. Survival analyses using Kaplan-Meier and MTCI databases revealed that lower *HOXB2* expression was significantly associated with poor overall survival (OS) in patients with breast cancer (Fig. S2a, b). Nevertheless, the expression levels of *HOXB3* were not correlated with patient prognosis, suggesting that HOXB2 is a pivotal component in the association of TNBC-related phenotypes (Fig. S2c, d). Real-time qPCR and western blot analyses confirmed the lower expression levels of HOXB2 in TNBC than in non-TNBC cell lines (**Fig. 1d, e**), further indicating a role for HOXB2 in tumor aggressiveness.

### HOXB2 attenuates aggressive phenotypes and EMT traits in breast cancer cells

**Figure 2:**
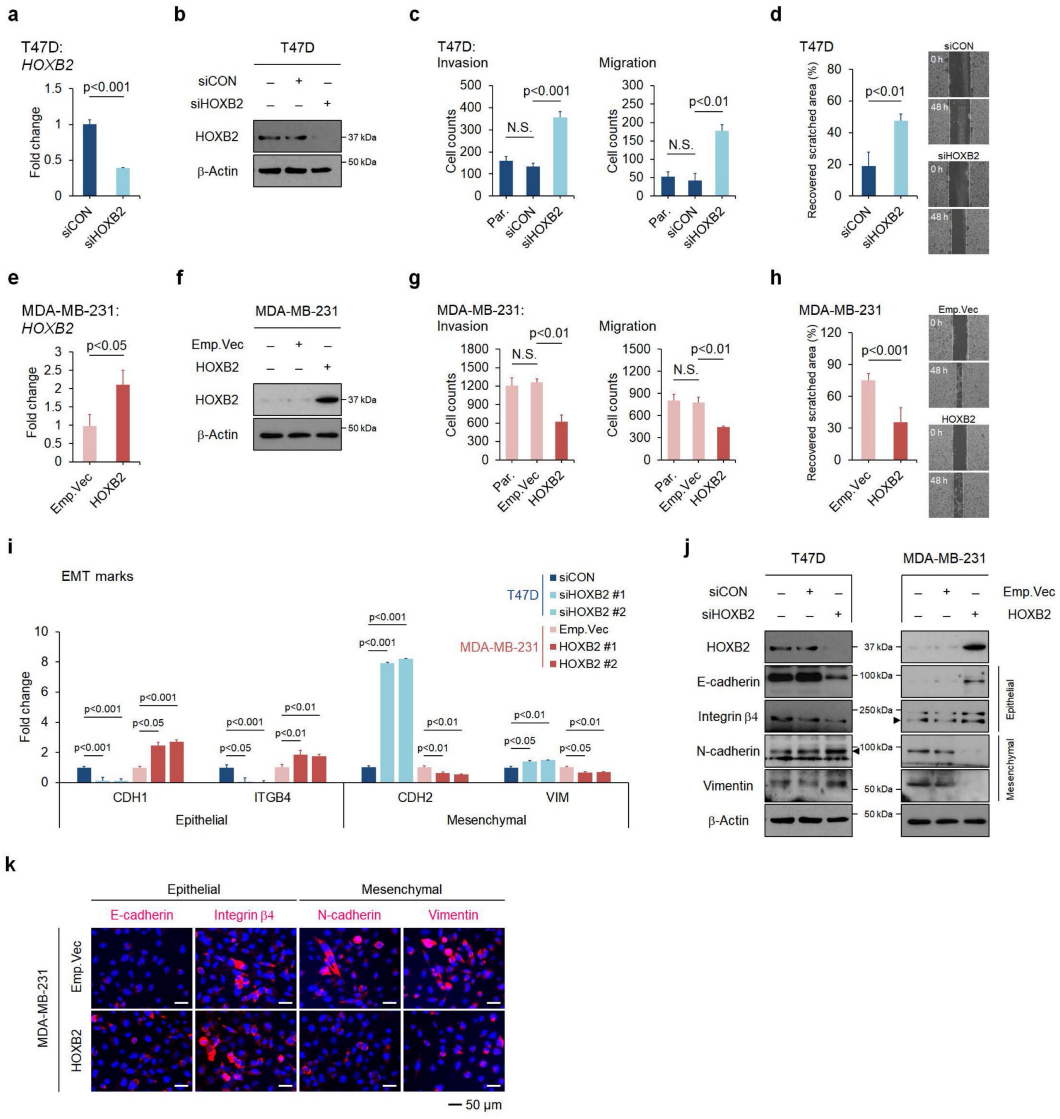
HOXB2 attenuates EMT traits in breast cancer cells. **a, b** mRNA (**a**) and protein (**b**) levels of knocked-down HOXB2 in T47D breast cancer cells. Two types of siRNAs were used in the HOXB2 knockdown experiment, and representative results are shown in the main figure. **c** Transwell assays of T47D cells transfected with siCON or siHOXB2. The invaded (left panel) and migrated (right panel) cells were stained with DAPI and counted. **d** Migration assay of T47D cells transfected with siCON or siHOXB2. **e, f** mRNA (**e**) and protein (**f**) levels of overexpressed HOXB2 in MDA-MB-231 breast cancer cells. **g** Transwell assays of MDA-MB-231 cells transfected with empty vector or HOXB2. The invaded (left panel) and migrated (right panel) cells were stained with DAPI and counted. **h** Migration assay of MDA-MB-231 cells transfected with empty vector or HOXB2. **i, j** qPCR (**i**) and western blotting (**j**) were performed to examine the expression levels of epithelial or mesenchymal markers in T47D cells transfected with siHOXB2 or MDA-MB-231 cells overexpressing HOXB2. **k** Immunofluorescence confocal microscopy of epithelial (E-cadherin and Integrin β4) and mesenchymal (N-cadherin and vimentin) markers in MDA-MB-231 cells overexpressing empty vector or HOXB2. Nuclei were stained with DAPI (blue).

To assess the functional role of HOXB2 in breast cancer progression, HOXB2 was knocked down in T47D ER^+^ breast cancer cell lines using siRNAs (**Fig. 2a, b**), followed by trans-well invasion and migration assays. HOXB2 knockdown significantly enhanced the invasive and migratory abilities of T47D cells (**Fig. 2c** and Fig. S3a). The scratch-migratory assay also revealed the migratory ability of HOXB2-knocked down T47D (T47D:siHOXB2) cells was significantly increased (**Fig. 2d**). In contrast, when HOXB2 was overexpressed in MDA-MB-231 TNBC cells (**Fig. 2e, f**), the invasion and migration capabilities were reduced (**Fig. 2g** and Fig. S3f). Furthermore, MDA-MB-231:HOXB2 cells were unable to close the scratched area as rapidly as the control cells (**Fig. 2h**).

To determine the effects of HOXB2 on EMT in breast cancer, the expression of EMT markers such as *CDH1*,*ITGB4, CDH2* and *VIM* were analyzed. *CDH1* and *ITGB4* epithelial markers were downregulated in the absence of HOXB2 in T47D cells and upregulated when HOXB2 was overexpressed in MDA-MB-231 cells. In contrast, mesenchymal genes *CDH2* and *VIM* were upregulated in T47D:siHOXB2 cells and downregulated in MDA-MB-231:HOXB2 cells (**Fig. 2i**). Similarly, the protein levels of these EMT-related markers paralleled their corresponding gene expression patterns (**Fig. 2j**). Consistently, forced expression of HOXB2 in MDA-MB-231 cells resulted in significant epithelial marker induction and an evident reduction in the levels of mesenchymal markers (**Fig. 2k**). Together, these results suggest that HOXB2 functions as an important component in the regulation of EMT in breast cancer cells.

### HOXB-AS1 is a positive upstream regulator of HOXB2 expression in breast cancer

**Figure 3:**
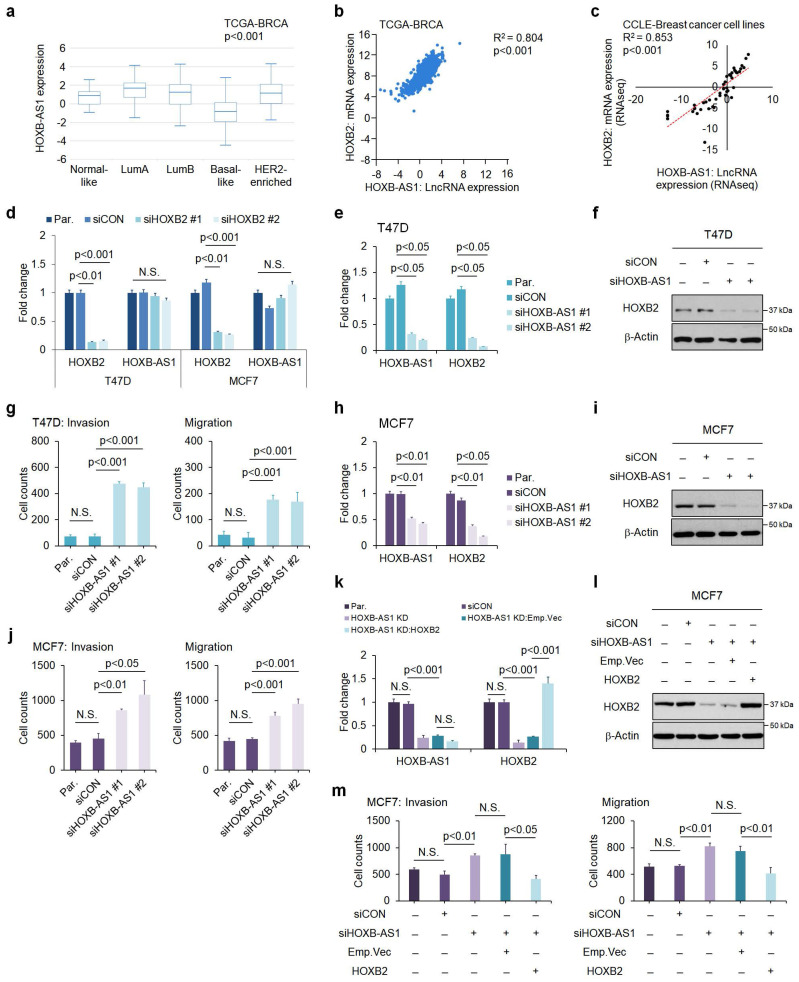
HOXB-AS1 is a positive upstream regulator of HOXB2 expression in breast cancer cell lines. **a** Analyses of TCGA dataset (Nature 2012) for the expression of HOXB-AS1 in different breast cancer subtypes. **b** Correlation curve between HOXB2 and HOXB-AS1 expression in the TCGA breast cancer tissue database. **c** Correlation curve between HOXB2 and HOXB-AS1 in CCLE breast cancer cell line database. **d** Real-time qPCR of HOXB2 and HOXB-AS1 in T47D and MCF7 cell lines transiently transfected with siHOXB2 for 24 hrs. Two types of siRNAs were used in the HOXB2 knockdown experiment, and representative results are shown in the main figure. **e** Real-time qPCR of HOXB2 and HOXB-AS1 in T47D cells transiently transfected with siHOXB-AS1 for 24 hrs. Two types of siRNAs were used in the HOXB-AS1 knockdown experiment. **f** Western blotting of HOXB2 in T47D cells transiently transfected with siHOXB-AS1 for 24 hrs. Two types of siRNAs were used in the HOXB-AS1 knockdown experiment. **g** Transwell assays of T47D cells transfected with siCON or siHOXB-AS1. The invaded (left panel) and migrated (right panel) cells were stained with DAPI and counted. Two types of siRNAs were used in the HOXB-AS1 knockdown experiment. **h** Real-time qPCR of HOXB2 and HOXB-AS1 in MCF7 cells transiently transfected with siHOXB-AS1 for 24 hrs. Two types of siRNAs were used in the HOXB-AS1 knockdown experiment. **i** Western blotting of HOXB2 in MCF7 cells transiently transfected with siHOXB-AS1 for 24 hrs. Two types of siRNAs were used in the HOXB-AS1 knockdown experiment. **j** Transwell assays of MCF7 cells transfected with siCON or siHOXB-AS1. Two types of siRNAs were used in the HOXB-AS1 knockdown experiment. The invaded (left panel) and migrated (right panel) cells were stained with DAPI and counted. **k** mRNA expression levels of HOXB2 and HOXB-AS1 in MCF7, MCF7:siCON, MCF7:siHOXB-AS1, MCF7:HOXB-AS1 KD:Emp.Vec, and MCF7:HOXB-AS1 KD:HOXB2 cells. **l** Protein expression levels of HOXB2 in MCF7, MCF7:siCON, MCF7:siHOXB-AS1, MCF7:HOXB-AS1 KD:Emp.Vec, and MCF7:HOXB-AS1 KD:HOXB2 cells. **m** Transwell assays of parent MCF7, MCF7:siCON, MCF7:siHOXB-AS1, MCF7:HOXB-AS1 KD:Emp.Vec, and MCF7:HOXB-AS1 KD:HOXB2 cells. The invaded (left panel) and migrated (right panel) cells were stained with DAPI and counted.

To elucidate the regulatory mechanism behind HOXB2 downregulation, we explored potential upstream regulators of HOXB2. Recently, the role of lncRNAs as a transcriptional regulator of neighboring genes has been highlighted^28^. Within the HOXB cluster, there is HOXB-AS1, a lncRNA positioned between HOXB2 and HOXB3. We examined the expression level of HOXB-AS1 in various subtypes of breast cancer using publicly available *in silico* datasets (**Fig. 3a**). The HOXB-AS1 transcripts were significantly downregulated in patients with basal-like breast cancer with markedly lower expression of HOXB2 compared with other subtypes (**Fig. 3a**). Further, we identified the positive correlation between HOXB2 and HOXB-AS1 in breast cancer patients as well as cell lines using data retrieved from TANRIC and CCLE (**Fig. 3b, c**).

To delineate the association between HOXB2 and HOXB-AS1, we first knocked down HOXB2 in T47D and MCF7 cells, then examined their expression levels. siHOXB2 treatment successfully knocked down HOXB2, but the expression of HOXB-AS1 was unchanged (**Fig. 3d**). Conversely, we knocked down HOXB-AS1 in T47D cells, and observed a significant downregulation of HOXB2 compared to control cells (**Fig. 3e, f**). Similar results were also observed in MCF7 cells (**Fig. 3h, i**). Taken together, these results reveal that HOXB-AS1 is a positive upstream regulator of HOXB2 in breast cancer cell lines.

To further demonstrate whether aggressive phenotypes are induced by HOXB-AS1-mediated HOXB2 expression, we carried out invasion and migration assays following HOXB-AS1 knockdown (**Fig. 3g, j** and Fig. S4a). The invasion and migratory abilities were significantly enhanced by knocking down HOXB-AS1 in T47D and MCF7 cell lines (**Fig. 3g, j** and Fig. S4a). Additionally, invasion and migration assays in MCF7:siHOXB-AS1 cells revealed that the cells rarely invaded and migrated despite HOXB-AS1 knockdown, compared to the behavior of the parent or control cells (**Fig. 3k-m** and Fig. S4b). Collectively, it strongly suggests that the aggressive phenotype of breast cancer cells is developed through the HOXB-AS1/HOXB2 transcriptional axis.

### Enrichment of SMYD3 by HOXB-AS1 correlates with HOXB2 expression in breast cancer cells by mediating H3K4me3 status

**Figure 4:**
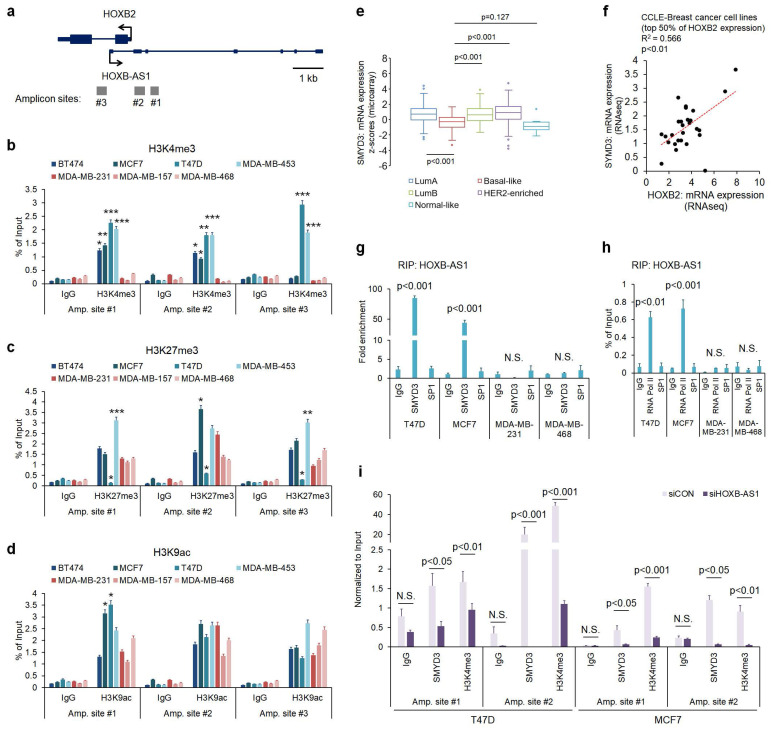
Induced enrichment of SMYD3 by HOXB-AS1 correlates with HOXB2 expression in breast cancer cell lines by mediating enrichment of H3K4me3. **a** Schematic depiction of the HOXB2 and HOXB-AS1 locus on human chromosome 17. Boxes represent exons, lines represent introns, and arrows show the direction of transcription. The gray bars represent the amplicon sites used in chromatin immunoprecipitation-qPCR (ChIP-qPCR). **b-d** ChIP-qPCR analysis of H3K4me3 (**b**), H3K27me3 (**c**), and H3K9ac (**d**) in various breast cancer cell lines. *P < 0.05, **P < 0.01, ***P < 0.001. **e** Analyses of TCGA dataset (Nature 2012) for the expression of SMYD3 in different breast cancer subtypes. **f** Correlation curve between HOXB2 and SMYD3 in CCLE breast cancer cell line database. **g** RIP-qPCR analysis for the interaction between HOXB-AS1 and SMYD3 in various breast cancer cell lines. SP1 was used as a negative control. **h** RIP-qPCR analysis for the interaction between HOXB-AS1 and RNA Pol II in various breast cancer cell lines. SP1 was used as a negative control. **i** ChIP-qPCR analysis of SMYD3 and H3K4me3 in HOXB-AS1-knockdown ER^+^ breast cancer cell lines. All experiments were performed in triplicate. Two types of siRNAs were used in the HOXB-AS1 knockdown experiment, and representative results are shown in the main figure.

To further understand the molecular mechanism behind HOXB-AS1-mediated HOXB2 transcription, we investigated histone modifications at the putative HOXB2 promoter region. We designed three amplicon sites (**Fig. 4a**) and analyzed H3K4me3, histone H3 lysine 27 trimethylation (H3K27me3), and histone H3 lysine 9 acetylation (H3K9ac) enrichment at those sites through ChIP-PCR. H3K4me3 marks, representative of active gene transcription, were significantly enriched in ER-positive breast cancer cells compared to TNBC cells at all three amplicon sites (**Fig. 4b**). On the other hand, H3K27me3 and H3K9ac enrichment did not show a significant difference between breast cancer subtypes (**Fig. 4c, d**). As a result, we investigated the reason behind the discrepant H3K4me3 marks at the putative HOXB2 promoter region. Of the various histone methyltransferases and histone demethylases, we discovered SMYD3 as a prospective factor. Using data retrieved from TCGA, we analyzed the expression of SMYD3 in normal breast and various breast cancer subtypes (**Fig. 4e**). Results showed that the expression of SMYD3 is particularly low in basal-like breast cancer compared to other subtypes. Under these circumstances, we hypothesized that due to the decreased amount of SMYD3 in TNBC, the enrichment of H3K4me3 at the HOXB2 promoter is prevented, ultimately leading to the downregulation of HOXB2.

Accordingly, we explored the relationship between the expression of HOXB2 and SMYD3 in breast cancer cell lines using data retrieved from CCLE dataset, and found a positive correlation (**Fig. 4f**). Consequently, we surmised that HOXB-AS1 plays a role as a guide molecule for SMYD3 to deposit H3K4me3 marks at the HOXB2 promoter. To illuminate the link between HOXB-AS1 and SMYD3, we performed RIP assay using HOXB-AS1 RNA as a bait and found that in non-TNBC cells, there is a direct interaction between HOXB-AS1 and SMYD3, but none could be observed in TNBC cells (**Fig. 4g**). We also verified that HOXB-AS1 forms a complex with RNA Pol II in recruiting SMYD3 (**Fig. 4h**). Finally, to confirm whether the binding of SMYD3, and the deposition of H3K4me3 at the HOXB2 promoter is truly mediated by HOXB-AS1, we knocked down HOXB-AS1 in ER-positive breast cancer cells and performed ChIP-PCR assay. We validated that the HOXB-AS1 knockdown markedly impeded the binding of SMYD3 and deposition of H3K4me3 at the HOXB2 promoter (**Fig. 4i**).

### Genome-wide identification of HOXB2 transcriptional targets in breast cancer

**Figure 5:**
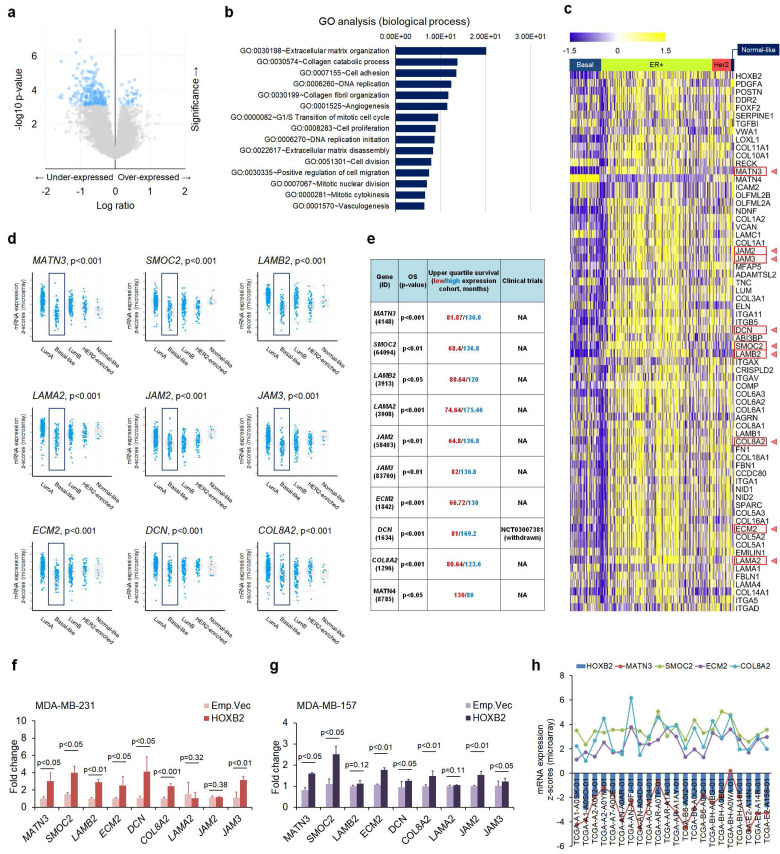
ECM organization molecules are involved in HOXB2-associated breast cancer progression. **a** Volcano plot for gene expression from the microarray generated by comparing paired HOXB2-altered and -unaltered breast cancer tissues from TCGA dataset. The blue dots indicate genes with significantly decreased expression (in the under-expressed part) and increased expression (in the over-expressed part) in the patient tissues with HOXB2 alteration. **b** GO analysis of HOXB2 dysregulation in TCGA dataset. The bar graph shows significant top enrichment scores. **c** Heatmap of microarray gene expression fold change from the ECM organization-associated genes in GO analysis (**b**). Each column is denoted as one patient. **d, e** Analyses of TCGA dataset (**d**) and Kaplan-Meier survival (**e**) for the expression of nine ECM organization genes in different subtypes of breast cancer. **f, g** Expression levels of nine ECM organization genes in MDA-MB-231:HOXB2 (**f**) and MDA-MB-157:HOXB2 (**g**) cells. **h** Positive correlation between HOXB2, MATN3, SMOC2, ECM2, and COL8A2 in patients with HOXB2-altered basal-like subtype breast cancer.

Next, to search the transcriptional targets of HOXB2 in TNBC, we analyzed genes that were expressed in similar patterns as *HOXB2* using cBioPortal (**Fig. 5a**). Using a 2-fold change cut-off, genes that were dysregulated together with *HOXB2* were mostly associated with ECM organization, collagen processing, and cell adhesion (**Fig. 5b**). The expression of the genes among different breast cancer subtypes were analyzed using a heatmap, and 67 genes were identified to have differential expression between TNBC and other subtypes (**Fig. 5c**). Of these, *MATN3*, *SMOC2*, *LAMB2*, *LAMA2*, *JAM2*, *JAM3*, *ECM2*, *DCN*, and *COL8A2* were exclusively downregulated in TNBC compared to ER^+^ breast cancer, and their expressions were significantly downregulated in basal-like breast cancers compared to normal-like, ER^+^, and/or HER2^+^ breast cancers (**Fig. 5d**). Moreover, low expression of these nine genes was also strongly correlated with poor OS in breast cancer patients (**Fig. 5e**).

To explore the interaction between HOXB2 and the nine genes, RT-qPCR was performed in MDA-MB-231 and MDA-MB-157 TNBC cells overexpressing an empty vector or HOXB2. *MATN3*, *SMOC2*, *ECM2*, and *COL8A2* were significantly upregulated when HOXB2 was overexpressed (**Fig. 5f, g**). Additionally, these genes were significantly downregulated in T47D:siHOXB2 cells (Fig. S5a). Consistent with this, *MATN3*, *SMOC2*, *ECM2*, and *COL8A2* expressions, in general, positively correlated with *HOXB2* expression in patients with TNBC exhibiting *HOXB2* alteration (**Fig. 5h**). However, the expression of other factors (*LAMB2*, *DCN*, *LAMA2*, *JAM2*, and *JAM3*) related to ECM organization was not associated with *HOXB2* expression (Fig. S5b).

### MATN3 and ECM2 are transcriptionally regulated by HOXB2 and are associated with aggressive phenotypes in TNBC

**Figure 6:**
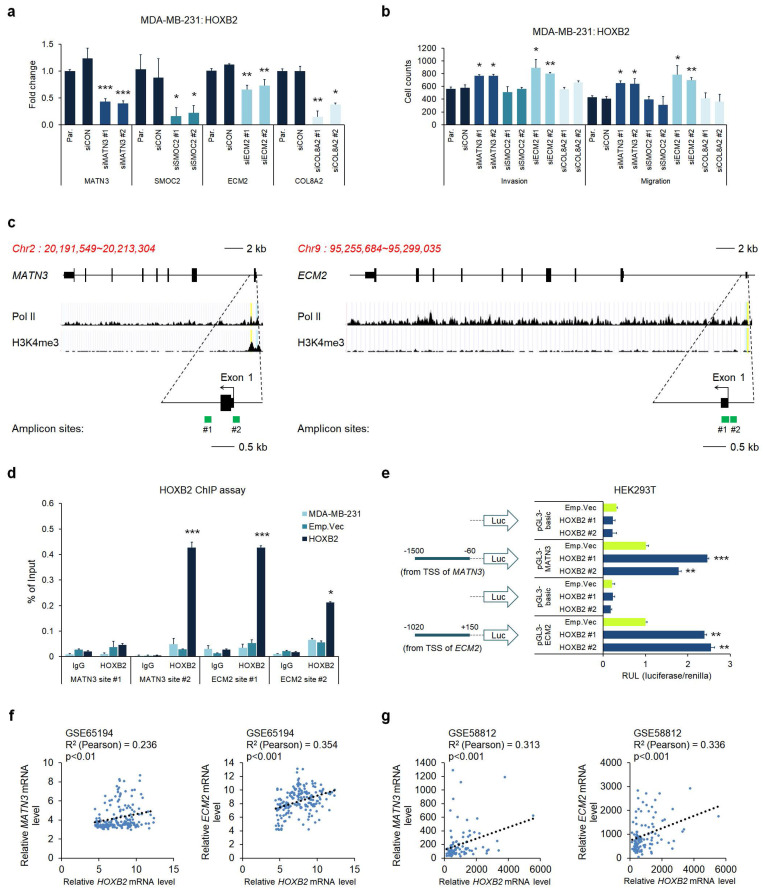
MATN3 and ECM2 are transcriptionally regulated by HOXB2 and are associated with aggressive phenotypes in TNBC. **a** Expression levels of *MATN3*, *SMOC2*, *ECM2*, and *COL8A2* in MDA-MB-231:HOXB2 cells transfected with siRNAs for each target gene. *P < 0.05, **P < 0.01, ***P < 0.001. **b** Transwell assays of MDA-MB-231:HOXB2 cells transfected with siCON or siMATN3, siSMOC2, siECM2, and siCOL8A2. The invaded (lower left panel) and migrated (lower right panel) cells were stained with DAPI and counted. *P < 0.05, **P < 0.01. **c** Schematic illustration of the predicted binding sites for HOXB2 in the indicated *MATN3* (left panel) and *ECM2* (right panel) promoter regions. The green squares represent the RT-qPCR amplicon sites. **d** ChIP analysis of the enrichment of HOXB2 at the *MATN3* and *ECM2* promoter regions. IgG was used as a negative control. *P < 0.05, ***P < 0.001. **e** Schematic illustration of the *MATN3* and *ECM2* promoter regions cloned into the pGL3 luciferase reporter plasmid (left panel). Quantification of the luciferase activity of the *MATN3* and *ECM2* promoter reporter was examined in HEK293T cells (right panel). **P < 0.01, ***P < 0.001. **f, g** Analysis of public datasets GSE65194 (**f**) and GSE58812 (**g**) for the expression of *MATN3*, *ECM2*, and *HOXB2*. The relative mRNA levels of *MATN3* and *ECM2* are plotted against those of *HOXB2*.

Considering the potential of HOXB2 as a transcription factor, we hypothesized that HOXB2 could constitute an upstream regulator of *MATN3*, *SMOC2*, *ECM2*, and *COL8A2*, leading to aggressive TNBC characteristics. To test this hypothesis, we performed RT-qPCR in the MDA-MB-231:HOXB2 cells transiently transfected with siRNAs targeting *MATN3*, *SMOC2*, *ECM2*, or *COL8A2*. Despite HOXB2 overexpression, the siRNAs successfully knocked down each downstream target gene (**Fig. 6a**). Invasion and migration assays in the MDA-MB-231:HOXB2 cells knocked down with siMATN3 or siECM2 showed that the cells readily invaded and migrated despite HOXB2 overexpression, compared to the behavior of the parent or control cells (**Fig. 6b** and Fig. S6). However, SMOC2 and COL8A2 knockdown did not significantly alter the invasion and/or migration abilities of the cells (**Fig. 6b**).

Given that MATN3, ECM2, and HOXB2 expressions were correlated, we further explored their functional relationships. The putative promoter regions of *MATN3* and *ECM2* were identified by RNA pol II and H3K4me3 enrichment. To investigate whether HOXB2 functions as a transcription factor, amplicon sites on the putative promoter were designed for ChIP experiments (**Fig. 6c**). Quantitative ChIP analysis on the *MATN3* and *ECM2* promoter regions with antibodies against HOXB2 in MDA-MB-231:HOXB2 cells revealed that HOXB2 is capable of binding to the promoters of the target genes to regulate their expression (**Fig. 6d**). To further support the function of HOXB2 as a transcriptional regulator of MATN3 and ECM2, a luciferase promoter assay was performed. As expected, HOXB2 overexpression led to an increase in the promoter activity of *MATN3* and *ECM2* compared to that in the parent and control HEK293T cells (**Fig. 6e**).

As HOXB2, MATN3, and ECM2 expressions positively correlated in breast cancer cells, we re-analyzed data from patient samples in publicly available clinical datasets of GSE65194 and GSE58812. HOXB2 expression strongly positively correlated with that of MATN3 and ECM2 in both datasets, again supporting our findings that MATN3 and ECM2 are transcriptionally regulated by HOXB2 (**Fig. 6f, g**).

### HOXB2 overexpression attenuates TNBC metastasis *in vivo*

**Figure 7:**
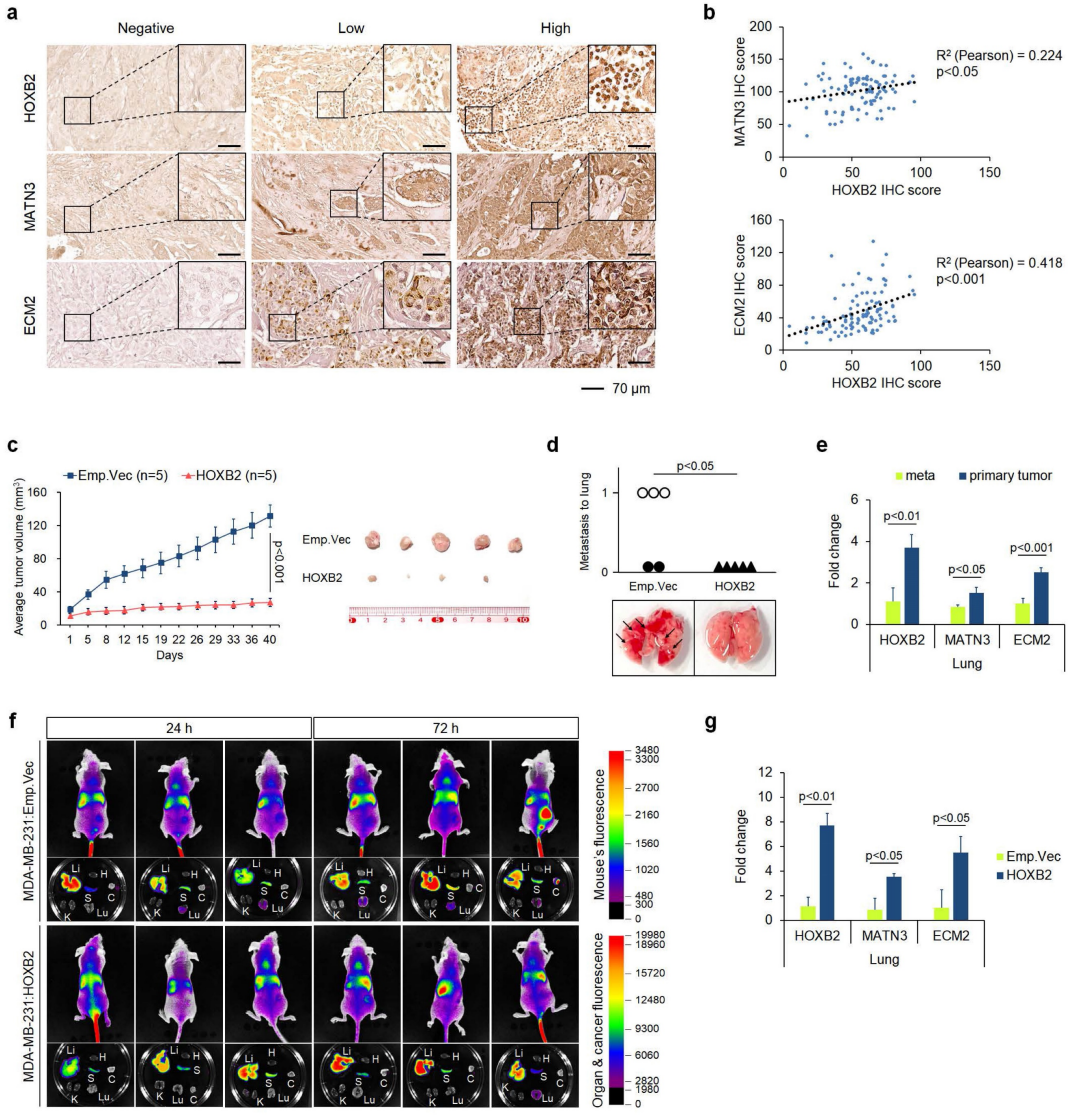
Overexpression of HOXB2 attenuates TNBC metastasis *in vivo*. **a** Representative IHC staining images of the HOXB2, MATN3, and ECM2 in breast cancer samples (n = 97). Left panels show negative signals of each protein; middle panels show low signals of each protein; right panels show high signals of each protein. Scale bar = 70 μm. **b** Positive correlation between HOXB2 and MATN3 (upper graph) or HOXB2 and ECM2 (lower graph). **c** HOXB2 overexpression reduces tumor growth in MDA-MB-231 xenograft mouse models. Left panel: tumor growth calculated and compared between each time point. Right panel: resected tumors from mice injected with MDA-MB-231:Emp.Vec, and MDA-MB-231:HOXB2 cells. For *in vivo* xenograft experiments, 5 mice per cell group were used. **d** Each circle (injection of control cells) and triangle (injection of HOXB2-overexpressing cells) represents an individual lung, and empty circles indicate the tumor has metastasized and developed in the lung. Lower panel shows pictures of representative lungs collected for each condition. Arrows indicate tumors formed at the lung. **e** RNA expression levels of *HOXB2*, *MATN3*, and *ECM2* in metastasized tumors in the lung and primary tumors. **f** HOXB2 overexpression reduces tumor metastasis in MDA-MB-231 xenograft mouse models. Tumor metastasis evaluated at different time points and compared between mice injected with MDA-MB-231:Emp.Vec, and MDA-MB-231:HOXB2 cells. Li: liver, H: heart, S: spleen, K: kidney, Lu: lung, C: cancer. For *in vivo* xenograft experiments, 6 mice per cell group were used. **g** Expression levels of *HOXB2*, *MATN3*, and *ECM2* in lung tissues from mice injected with MDA-MB-231:Emp.Vec, and MDA-MB-231:HOXB2 cells.

To further confirm the therapeutic efficacy of HOXB2/MATN3 or HOXB2/ECM2 transcriptional axis, we performed IHC for HOXB2, MATN3, and ECM2 in-house with 100 human tissue samples of patients with breast cancer (**Fig. 7a**). The IHC scores of HOXB2, MATN3, and ECM2 were compared by Pearson correlation analysis to evaluate any correlation (**Fig. 7b**).

Both MATN3 (**Fig. 7b**; upper graph) and ECM2 (**Fig. 7b**; lower graph) expressions were positively correlated with HOXB2 expression in the human breast cancer tissues. Moreover, clinicopathological features including age, hormone receptor status, histological grade, clinical stage, and Ki67 score were examined depending on the signal intensity of HOXB2, MATN3, and ECM2 categorized into negative/low and high status (**Table 1**). HOXB2 expression significantly positively correlated with hormone receptor expression and HOXB2-negative/low expression was observed in patients with TNBC. Moreover, Ki67, a marker of cell proliferation, also significantly negatively correlated with HOXB2 expression (**Table 1**). MATN3 expression was also positively correlated with hormone receptor expression and negatively with clinical stage and Ki67 expression. ECM2 expression correlated with hormone receptor expressions (**Table 1**). These findings support that HOXB2 is invariably associated with TNBC and its features. Nonetheless, a statistically significant relationship between HOXB2 and lymph node metastasis in patients with breast cancer could not be discerned from our in-house analysis. Therefore, we utilized the Metastatic Breast Cancer Project data to determine the degree of metastasis depending on HOXB2 expression. We found that breast cancers with metastasis to regional lymph nodes, the brain and the central nervous system (CNS), and the liver have low levels of HOXB2 (Fig. S7a**-**c). When HOXB2 expression was low, metastasis occurred during treatment regardless of the pre-existing or newly formed metastatic tumors. These data were consistent with our results from the *in silico* and *in vitro* studies, suggesting that HOXB2 plays an important role in breast cancer invasion, migration, and ultimately metastasis in patients with TNBC.

To assess whether cancer progression is regulated by HOXB2 *in vivo*, we injected MDA-MB-231:Emp.Vec and MDA-MB-231:HOXB2 cells subcutaneously into the mammary fat pads of BALB/c nude mice. Primary tumor growth in each mouse was monitored for 8 weeks, after which the animals were sacrificed to determine the degree of the lung metastasis. Control mice injected with MDA-MB-231:Emp.Vec exhibited significant tumor growth, whereas mice injected with MDA-MB-231:HOXB2 cells could not form tumors of tangible size. On day 58, the mean tumor volume was 131.82 ± 13.18 mm^3^ and 27.29 ± 4.8 mm^3^ for the control and HOXB2-overexpressing groups, respectively (**Fig. 7c**). Notably, metastasized lung tumors were found in three of five MDA-MB-231:Emp.Vec-injected mice; however, no metastasized tumors were observed in MDA-MB-231:HOXB2-injected mice, demonstrating that HOXB2 overexpression protects mice from lung metastasis (**Fig. 7d**). In addition, primary and lung-metastasized tumors were collected and sampled to explore the expression levels of *HOXB2* and its downstream genes using RT-qPCR. In particular, the expression of *HOXB2*, *MATN3*, and *ECM2* was significantly suppressed in metastatic tumors compared to that in primary tumors (**Fig. 7e**).

Additionally, to assess whether metastasis is inhibited by the presence of HOXB2 *in vivo*, we injected MDA-MB-231:Emp.Vec and MDA-MB-231:HOXB2 cells intravenously into the tail vein of BALB/c nude mice. Tumor metastasis in each mouse was monitored for 3 days, after which the animals were sacrificed to determine the degree of the various organ metastasis of cancer cells. Tail vein injection experiments with MDA-MB-231:HOXB2 showed cells rarely metastasizing to the lungs, compared to that of control cells. In empty vector mice, signals were observed in the lungs, spleens, and liver starting at 24 hrs. On the other hand, HOXB2 overexpressing mice started to show signals in the lung at 3 days post injection. (**Fig. 7f**). Further, analyses of HOXB2 and downstream gene expressions in lung tissues collected revealed a significant increase in the expression of HOXB2, MATN3, and ECM2 in MDA-MB-231:HOXB2 lung tissues compared to that in MDA-MB-231:Emp.Vec lung tissues. (**Fig. 7g**).

### HOXB2 and MATN3/ECM2 are downregulated in multiple carcinomas and their downregulation correlates with worse survival

**Figure 8:**
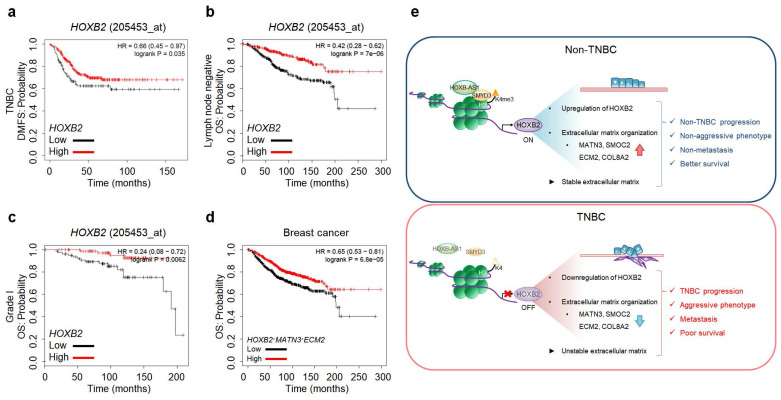
HOXB2, MATN3, and ECM2 are robust clinical markers for patients with breast cancers. **a-c** Kaplan-Meier DMFS analyses comparing high and low levels of *HOXB2* in TNBC (**a**), lymph node-negative (**b**), Grade I (**c**) cancers. **d** OS curves of total patients with breast cancer exhibiting high or low *HOXB2*/*MATN3*/*ECM2* expression. **e** Schematic model of the coordinated role of HOXB2 in TNBC tumorigenesis. In non-TNBC, HOXB-AS1 acts as a guide molecule for SMYD3 to deposit H3K4me3 marks at the HOXB2 promoter, leading to the transcriptional activation of HOXB2. This leads to an epithelial phenotype and increases the expression of extracellular matrix organization molecules. Consequently, non-TNBC exhibits significantly less aggressive and less metastatic phenotypes, resulting in better survival rates. Conversely, decreased enrichment of HOXB-AS1/SMYD3 complex inhibits HOXB2 expression by mediating H3K4me3 status in TNBC. This induces a mesenchymal phenotype and reduces the expression of extracellular matrix organization molecules. Consequently, TNBC exhibits aggressive and metastatic phenotypes, resulting in poor survival rates.

As HOXB2 directly regulates MATN3 and ECM2 at the transcriptional level and induces their downregulation in TNBC cells, leading to aggressive EMT-associated phenotypes and ultimately metastasis, we next evaluated Kaplan-Meier survival curves to evaluate the clinical relevance of our findings. Low expression of HOXB2 was associated with poor DMFS in TNBC (**Fig. 8a**). Moreover, poor OS associated with low HOXB2 expression was observed in patients with lymph node-negative breast cancer (**Fig. 8b**). This supports the notion that HOXB2 when expressed at low levels in early-stage cancer (lymph node-negative), could modulate the invasion and migration of TNBC cells and ultimately lead to metastatic cancer. Similarly, low HOXB2 expression was significantly associated with poor OS in patients with Grade I breast cancer (**Fig. 8c**). Meanwhile, low expression of HOXB2 was associated with poor OS in both patients with ER^+^ or PR^+^ breast cancer (Fig. S8a, b). As a result, the correlation between the expression of HOXB2 and ESR1 and/or PGR was analyzed (Fig. S8c, d). There was indeed a positive correlation between HOXB2 and both ESR1 and PGR, allowing the conclusion to be drawn that low expression of HOXB2 could lead to the transition of ER^+^ and/or PR^+^ breast cancers to express less hormone receptors, and display more aggressive phenotypes like TNBC.

Moreover, poor OS in patients with breast cancer was significantly associated with the concurrent downregulation of HOXB2, MATN3, and ECM2 (**Fig. 8d**). The same trend of worse prognosis was observed in patients with lung cancer, renal clear cell carcinoma, or hepatocellular carcinoma exhibiting low HOXB2 and/or low HOXB2/MATN3/ECM2 expression (Fig. S8e-g). These results suggest that the downregulation of HOXB2 and its downstream factors, MATN3 and ECM2, is not only involved in the malignant progression of breast cancer but also in that of other solid tumors.

## Discussion

As *HOX* genes are crucial during development, their dysregulation often contributes to tumor growth, progression, and metastasis^4^, ^29^. Previous studies have reported that HOXB2 functions as either a tumor suppressor or an oncogene in several types of tumors. As an oncogene, its role in regulating cell proliferation, invasion, and migration has been reported in glioblastoma, lung, ovarian, and colorectal cancers^5^, ^8^, ^9^, ^30^. In contrast, here, we identified that the level of HOXB2 is reduced in TNBC. In non-TNBC, HOXB-AS1 promotes HOXB2 expression by acting as a guide molecule for SMYD3 to attach the H3K4me3 mark to the HOXB2 promoter region. However, due to the low expression of HOXB-AS1 in TNBC, the guide role for SMYD3 was insufficient, resulting in decreased HOXB2 expression. Additionally, our findings suggest that the lack of HOXB2 subsequently downregulates MATN3 and the ECM2 downstream transcriptional network, leading to aggressive cancer progression, EMT-like characteristics, and poor prognosis (**Fig. 8e**). We demonstrated that forced expression of HOXB2 in TNBC cells suppresses invasive and migratory characteristics *in vitro* along with the suppression of tumor growth and metastasis *in vivo*. Moreover, HOXB2 downregulation was associated with the poor OS not only in patients with breast cancer but also in patients with other solid tumors. In this context, HOXB2 has also been demonstrated to constitute a tumor suppressor in mammary adenocarcinoma tumors by negatively regulating tumor progression^5^, ^10^. Especially, in the previous study, Boimel et al performed an *in vivo* shRNA screen and were able to determine the changes in proportion of the knockdown cell lines after selection for *in vivo* tumor growth as well as seeding and growth in lung metastases after tail vein injection. As a result, the authors confirmed that HOXB2 has been reported to act as a negative tumor growth regulator and reduce the proliferation of breast adenocarcinoma tumors^10^. In addition, this research not only confirmed that overexpression of HOXB2 was associated with better prognostic results through KM plot analysis, but also verified that HOXB2 was significantly underexpressed in higher grade breast cancer. Further, as a result of grading analysis for HOXB2 in breast cancer using Oncomine, HOXB2 expression was found to be significantly lower in grade 3 tumors in 7 out of 14 existing microarray studies^10^. Further to these data, our current study revealed that the lack of HOXB2 enhances the invasive and migratory characteristics of TNBC and promotes EMT by reducing ECM organization.

The process of EMT begins with biochemical changes in the epithelial cells that enable them to become mesenchymal-like^31^. Network analysis revealed that HOXB2 is associated with a cluster of factors related to ECM organization consisting of MATN3 and ECM2. MATN3 is an ECM protein that is present at high levels during chondrogenesis^23^, ^32^, ^33^ but not limited to cartilage^32^, whereas ECM2 is an extracellular glycoprotein that is important in cell growth factor signaling, cell migration, and angiogenesis^25^, ^34^. During EMT, migratory capacity and invasiveness are enhanced, and the production of ECM components, such as MATN3 and ECM2, is greatly decreased. As a final step, the basement membrane is degraded once it interacts with the epithelial cells so that the newly formed mesenchymal cells migrate away from the epithelial layer^35^. The key findings of the present study are the importance of the homeodomain protein HOXB2 and its potential as a regulator of these ECM components as downstream targets by binding to their respective promoter regions. Thus, the absence of HOXB2 in TNBC disrupts the formation of the ECM and the basement membrane, ultimately permitting aggressive characteristics.

Epigenetic modifications, such as DNA methylation and histone modifications, play important roles in various cellular processes during the development and progression of numerous cancers^36^. In particular, histone modifications are implicated in many cancers by controlling chromatin structure and gene transcriptions^37^. H3K4me3 is an important active marker known to promote gene transcription^38^. At histone H3, lysine residues can be mono-, di-, and trimethylated by writers or demethylated by erasers^39^. SMYD2 is a member of the SMYD family, which acts as a writer^39^. SMYD2 is involved in the methylation of H3K4 and H3K36 and non-histone proteins, such as P53, HSP90, ERα, PARP1, MAPKAPK3, and PTEN^40^. In particular, it is demonstrated that SMYD2 has an important role in TNBC progression by directly acting as a methyltransferase of EZH2 at lysine 307^41^. Additionally, SMYD2 promotes TNBC progression through post-translational regulation of STAT3 and NF-kB, in that increased SMYD2 activates STAT3 and NF-kB and exerts a synergistic effect on the increase of TNBC cell proliferation and survival^42^. As one of the H3K4 tri-methyl transferases, SMYD3 also has been shown to regulate proliferation, migration, and invasion abilities in numerous carcinomas, such as colon, stomach, melanoma, and liver^43^. Contrary to SMYD2, SMYD3 was downregulated in TNBC patients and negatively correlated with advanced scarff-bloom-richardson grades^17^. In previous study, we observed a lower enrichment of SMYD3/H3K4me3 at the HOXB2 promoter region, consistent with decreased HOXB2 expression in TNBC. These results are important in that there is only observation showing the dysregulation of SMYD3 in TNBC, and we showed a novel mechanism by which HOXB2 is regulated via SMYD3.

Recent evidence highlighted lncRNAs as potential biomarkers and therapeutic targets in human cancers. Among various types of lncRNAs, antisense lncRNAs located adjacent to coding transcripts have been shown to regulate neighboring coding genes^44^. In particular, numerous lncRNAs are embedded in the HOX cluster (HOX-lncRNAs) in an antisense manner. Several HOX-lncRNAs have been shown to impact tumorigenesis and cancer progression in many cancers^45^. The lncRNA HOTTIP (HOXA transcript at the distal tip), located near HOXA13, is implicated in the progression of hepatocellular carcinoma (HCC) and gastric cancer by regulating HOXA13^45^. According to Quagliata et al., the HOTTIP/HOXA13 axis is regulated by a bi-directional loop between the two genes in HCC^46^. Additionally, HOTTIP is involved in the tumorigenesis and metastasis of esophageal squamous carcinoma cells by mediating miR-30b^47^. HOTAIR (HOX antisense intergenic RNA) which neighbors HOXC11 and HOXC12 has been demonstrated to be co-expressed with HOXC12, and downregulated in ER- and basal-like breast cancers compared to other BC subtypes^48^. HOTAIR interacts with various microRNAs and regulates gene expression by binding to polycomb repressive complexes 2 inducing H3K27me3, resulting in a pro-oncogenic phenotype in gliomas^49^. In previous study, we demonstrated that HOTAIRM1 (HOXA transcript antisense RNA, myeloid-specific 1), located between HOXA1 and HOXA2, has an important role in acquired tamoxifen resistance by regulating HOXA1 transcription^27^. Therefore, in this study, we focused on the mechanism by which HOXB-AS1 regulates its neighboring gene, HOXB2, in non-TNBC cells. Through *in silico* and *in vitro* studies, we found that HOXB-AS1/HOXB2 is downregulated in TNBC compared to other BC subtypes. These findings highlight the role of HOXB-AS1 as an upstream regulator of HOXB2 and put forward HOXB-AS1 as a potential therapeutic target or biomarker for TNBC lacking targeted therapy. Since the relationship between coding genes and lncRNAs in tumorigenesis has been reported to be mediated by competing microRNAs, further investigation of the association of numerous miRNAs with the HOXB-AS1/SMYD3/HOXB2 axis might provide a further detailed mechanism with respect to TNBC tumorigenesis and metastasis.

## Conclusions

To our knowledge, this study is the first to reveal that HOXB2 plays a previously underappreciated role in TNBC and suggests that the selective targeting of HOXB2 may help advance therapeutic strategies and provide a new paradigm for treating patients with TNBC using non-invasive procedures.

## Supplementary Material

Supplementary figures and tables.

## Figures and Tables

**Table 1:** Clinicopathological features and HOXB2, MATN3, and ECM2 protein expression in patients with breast cancer (n=100).

	HOXB2 score		*P*-value	MATN3 score		*P*-value	ECM2 score		*P*-value
	Low	High		Low	High		Low	High	
Age (years)			0.335			0.236			0.475
≤ 50	20 (71.4%)	44 (61.1%)		21 (75%)	45 (62.5%)		20 (71.4%)	46 (63.9%)	
> 50	8 (28.6%)	28 (38.9%)		7 (25%)	27 (37.5%)		8 (28.6%)	26 (36.1%)	
ER status			**1.74E-05**			**0.001**			**0.004**
Negative	19 (67.9%)	16 (22.2%)		18 (64.3%)	17 (23.6%)		16 (57.1%)	19 (26.4%)	
Positive	9 (32.1%)	56 (77.8%)		10 (35.7%)	55 (76.4%)		12 (42.9%)	53 (73.6%)	
PR status			**0.001**			**0.001**			**0.031**
Negative	21 (75.0%)	26 (36.1%)		21 (75%)	26 (36.1%)		18 (64.3%)	29 (40.3%)	
Positive	7 (25.0%)	46 (63.9%)		7 (25%)	46 (63.9%)		10 (35.7%)	43 (59.7%)	
HER2 status			**0.013**			0.449			0.276
Negative	23 (82.1%)	40 (55.6%)		16 (57.1%)	47 (65.3%)		20 (71.4%)	43 (59.7%)	
Positive	5 (17.9%)	32 (44.4%)		12 (42.9%)	25 (34.7%)		8 (28.6%)	29 (40.3%)	
LN metastasis			0.715			0.383			0.096
Negative	20 (71.4%)	54 (75.0%)		19 (67.9%)	55 (76.4%)		24 (85.7%)	50 (69.4%)	
Positive	8 (28.6%)	18 (25.0%)		9 (32.1%)	17 (23.6%)		4 (14.3%)	22 (30.6%)	
Histologic grade			0.884			0.383			0.607
I, II	21 (75.0%)	55 (76.4%)		19 (67.9%)	55 (76.4%)		20 (71.4%)	55 (76.4%)	
III	7 (25.0%)	17 (23.6%)		9 (32.1%)	17 (23.6%)		8 (28.6%)	17 (23.6%)	
Stage			0.089			**0.039**			0.533
I - II	25 (89.3%)	53 (73.6%)		18 (64.3%)	60 (83.3%)		23 (82.1%)	55 (76.4%)	
III - IV	3 (10.7%)	19 (26.4%)		10 (35.7%)	12 (16.7%)		5 (17.9%)	17 (23.6%)	
Ki67			**2.67E-09**			**0.026**			0.368
≤ 20%	9 (32.1%)	65 (90.3%)		21 (75%)	66 (91.7%)		23 (82.1%)	64 (88.9%)	
> 20%	19 (67.9%)	7 (9.7%)		7 (25%)	6 (8.3%)		5 (17.9%)	8 (11.1%)	

## Abbreviations

BC: breast cancer
CCLE: cancer cell line encyclopedia
ChIP: chromatin immunoprecipitation
ECM: extracellular matrix
ECM2: extracellular matrix protein 2
EMT: epithelial-to-mesenchymal transition
ER: estrogen receptor
HER2: human epidermal growth factor receptor 2
HOXB-AS1: HOXB cluster antisense RNA 1
H3K4me3: histone H3 lysine 4 trimethylation
ICC: immunocytochemistry
LncRNAs: long non-coding RNAs
MATN3: matrillin 3
OS: overall survival
PR: progesterone receptor
RIP: RNA immunoprecipitation
SMYD3: SET and MYND domain-containing proteins 3
SPARC: secreted protein acidic and rich in cysteine
TMA-IHC: tissue microarray and immunohistochemistry
TNBC: triple-negative breast cancer

## References

[1] Foulkes WD, Smith IE, Reis-Filho JS (2010). Triple-negative breast cancer. N Engl J Med.

[2] Collignon J, Lousberg L, Schroeder H, Jerusalem G (2016). Triple-negative breast cancer: treatment challenges and solutions. Breast Cancer (Dove Med Press).

[3] Pearson JC, Lemons D, McGinnis W (2005). Modulating Hox gene functions during animal body patterning. Nat Rev Genet.

[4] Shah N, Sukumar S (2010). The Hox genes and their roles in oncogenesis. Nat Rev Cancer.

[5] Inamura K, Togashi Y, Ninomiya H, Shimoji T, Noda T, Ishikawa Y (2008). HOXB2, an adverse prognostic indicator for stage I lung adenocarcinomas, promotes invasion by transcriptional regulation of metastasis-related genes in HOP-62 non-small cell lung cancer cells. Anticancer Res.

[6] Gonzalez-Herrera A, Salgado-Bernabe M, Velazquez-Velazquez C, Salcedo-Vargas M, Andrade-Manzano A, Avila-Moreno F (2015). Increased expression of HOXB2 and HOXB13 proteins is associated with HPV infection and cervical cancer progression. Asian Pac J Cancer Prev.

[7] Xu F, Liu Z, Liu R, Lu C, Wang L, Mao W (2020). Epigenetic induction of tumor stemness via the lipopolysaccharide-TET3-HOXB2 signaling axis in esophageal squamous cell carcinoma. Cell Commun Signal.

[8] Yu HY, Pan SS (2020). MiR-202-5p suppressed cell proliferation, migration and invasion in ovarian cancer via regulating HOXB2. Eur Rev Med Pharmacol Sci.

[9] Chen X, Li LQ, Qiu X, Wu H (2019). Long non-coding RNA HOXB-AS1 promotes proliferation, migration and invasion of glioblastoma cells via HOXB-AS1/miR-885-3p/HOXB2 axis. Neoplasma.

[10] Boimel PJ, Cruz C, Segall JE (2011). A functional in vivo screen for regulators of tumor progression identifies HOXB2 as a regulator of tumor growth in breast cancer. Genomics.

[11] Novikova IV, Hennelly SP, Sanbonmatsu KY (2012). Sizing up long non-coding RNAs: do lncRNAs have secondary and tertiary structure?. Bioarchitecture.

[12] Liu D, Qiu M, Jiang L, Liu K (2020). Long Noncoding RNA HOXB-AS1 Is Upregulated in Endometrial Carcinoma and Sponged miR-149-3p to Upregulate Wnt10b. Technol Cancer Res Treat.

[13] Jaiswal D, Turniansky R, Moresco JJ, Ikram S, Ramaprasad G, Akinwole A (2020). Function of the MYND Domain and C-Terminal Region in Regulating the Subcellular Localization and Catalytic Activity of the SMYD Family Lysine Methyltransferase Set5. Mol Cell Biol.

[14] Tracy C, Warren JS, Szulik M, Wang L, Garcia J, Makaju A (2018). The Smyd Family of Methyltransferases: Role in Cardiac and Skeletal Muscle Physiology and Pathology. Curr Opin Physiol.

[15] Bernard BJ, Nigam N, Burkitt K, Saloura V (2021). SMYD3: a regulator of epigenetic and signaling pathways in cancer. Clin Epigenetics.

[16] Fenizia C, Bottino C, Corbetta S, Fittipaldi R, Floris P, Gaudenzi G (2019). SMYD3 promotes the epithelial-mesenchymal transition in breast cancer. Nucleic Acids Res.

[17] Song J, Liu Y, Chen Q, Yang J, Jiang Z, Zhang H (2019). Expression patterns and the prognostic value of the SMYD family members in human breast carcinoma using integrative bioinformatics analysis. Oncol Lett.

[18] Frantz C, Stewart KM, Weaver VM (2010). The extracellular matrix at a glance. J Cell Sci.

[19] Henke E, Nandigama R, Ergun S (2019). Extracellular Matrix in the Tumor Microenvironment and Its Impact on Cancer Therapy. Front Mol Biosci.

[20] Winkler J, Abisoye-Ogunniyan A, Metcalf KJ, Werb Z (2020). Concepts of extracellular matrix remodelling in tumour progression and metastasis. Nat Commun.

[21] Oskarsson T (2013). Extracellular matrix components in breast cancer progression and metastasis. Breast.

[22] Lu P, Takai K, Weaver VM, Werb Z (2011). Extracellular matrix degradation and remodeling in development and disease. Cold Spring Harb Perspect Biol.

[23] Klatt AR, Klinger G, Paul-Klausch B, Kuhn G, Renno JH, Wagener R (2009). Matrilin-3 activates the expression of osteoarthritis-associated genes in primary human chondrocytes. FEBS Lett.

[24] Yu Y, Chen Y, Ma J, Yu X, Yu G, Li Z (2016). SPARCL1 is a novel predictor of tumor recurrence and survival in hilar cholangiocarcinoma. Tumour Biol.

[25] Liu X, Zhao J, Luan X, Li S, Zhai J, Liu J (2020). SPARCL1 impedes trophoblast migration and invasion by down-regulating ERK phosphorylation and AP-1 production and altering EMT-related molecule expression. Placenta.

[26] Hur H, Lee JY, Yun HJ, Park BW, Kim MH (2014). Analysis of HOX gene expression patterns in human breast cancer. Mol Biotechnol.

[27] Kim CY, Oh JH, Lee JY, Kim MH (2020). The LncRNA HOTAIRM1 Promotes Tamoxifen Resistance by Mediating HOXA1 Expression in ER+ Breast Cancer Cells. J Cancer.

[28] Huarte M (2015). The emerging role of lncRNAs in cancer. Nat Med.

[29] Bhatlekar S, Fields JZ, Boman BM (2014). HOX genes and their role in the development of human cancers. J Mol Med (Berl).

[30] Li H, Zhu G, Xing Y, Zhu Y, Piao D (2020). miR-4324 functions as a tumor suppressor in colorectal cancer by targeting HOXB2. J Int Med Res.

[31] Dongre A, Weinberg RA (2019). New insights into the mechanisms of epithelial-mesenchymal transition and implications for cancer. Nat Rev Mol Cell Biol.

[32] Wu PL, He YF, Yao HH, Hu B (2018). Martrilin-3 (MATN3) Overexpression in Gastric Adenocarcinoma and its Prognostic Significance. Med Sci Monit.

[33] Lu XD, Liu YR, Zhang ZY (2020). Matrilin-3 alleviates extracellular matrix degradation of nucleus pulposus cells via induction of IL-1 receptor antagonist. Eur Rev Med Pharmacol Sci.

[34] Jakharia A, Borkakoty B, Singh S (2016). Expression of SPARC like protein 1 (SPARCL1), extracellular matrix-associated protein is down regulated in gastric adenocarcinoma. J Gastrointest Oncol.

[35] Scheau C, Badarau IA, Costache R, Caruntu C, Mihai GL, Didilescu AC (2019). The Role of Matrix Metalloproteinases in the Epithelial-Mesenchymal Transition of Hepatocellular Carcinoma. Anal Cell Pathol (Amst).

[36] Cheng Y, He C, Wang M, Ma X, Mo F, Yang S (2019). Targeting epigenetic regulators for cancer therapy: mechanisms and advances in clinical trials. Signal Transduct Target Ther.

[37] Zhao Z, Shilatifard A (2019). Epigenetic modifications of histones in cancer. Genome Biol.

[38] Lauberth SM, Nakayama T, Wu X, Ferris AL, Tang Z, Hughes SH (2013). H3K4me3 interactions with TAF3 regulate preinitiation complex assembly and selective gene activation. Cell.

[39] Hyun K, Jeon J, Park K, Kim J (2017). Writing, erasing and reading histone lysine methylations. Exp Mol Med.

[40] Weirich S, Schuhmacher MK, Kudithipudi S, Lungu C, Ferguson AD, Jeltsch A (2020). Analysis of the Substrate Specificity of the SMYD2 Protein Lysine Methyltransferase and Discovery of Novel Non-Histone Substrates. Chembiochem.

[41] Zeng Y, Qiu R, Yang Y, Gao T, Zheng Y, Huang W (2019). Regulation of EZH2 by SMYD2-Mediated Lysine Methylation Is Implicated in Tumorigenesis. Cell Rep.

[42] Li LX, Zhou JX, Calvet JP, Godwin AK, Jensen RA, Li X (2018). Lysine methyltransferase SMYD2 promotes triple negative breast cancer progression. Cell Death Dis.

[43] Bottino C, Peserico A, Simone C, Caretti G (2020). SMYD3: An Oncogenic Driver Targeting Epigenetic Regulation and Signaling Pathways. Cancers (Basel).

[44] Yu H, Rong L (2018). Emerging role of long non-coding RNA in the development of gastric cancer. World J Gastrointest Oncol.

[45] Wang Y, Dang Y, Liu J, Ouyang X (2016). The function of homeobox genes and lncRNAs in cancer. Oncol Lett.

[46] Chang S, Liu J, Guo S, He S, Qiu G, Lu J (2016). HOTTIP and HOXA13 are oncogenes associated with gastric cancer progression. Oncol Rep.

[47] Lin C, Wang Y, Wang Y, Zhang S, Yu L, Guo C (2017). Transcriptional and posttranscriptional regulation of HOXA13 by lncRNA HOTTIP facilitates tumorigenesis and metastasis in esophageal squamous carcinoma cells. Oncogene.

[48] Li C, Cui J, Zou L, Zhu L, Wei W (2020). Bioinformatics analysis of the expression of HOXC13 and its role in the prognosis of breast cancer. Oncol Lett.

[49] Angelopoulou E, Paudel YN, Piperi C (2020). Critical role of HOX transcript antisense intergenic RNA (HOTAIR) in gliomas. J Mol Med (Berl).

