# Supplementary material for: The homeoprotein HOXB2 limits triple-negative breast carcinogenesis via extracellular matrix remodeling
Source: Int J Biol Sci. 2024 Jan 20;20(3):1045–63. doi: 10.7150/ijbs.88837 (PMC10845296; doi:10.7150/ijbs.88837)
Supplement: Supplementary file 1 — Supplementary figures and tables. [file ijbsv20p1045s1.pdf]

Supplementary Information

The homeoprotein HOXB2 limits triple-negative breast carcinogenesis via extracellular matrix remodeling

Ji Hoon Oh[1], Clara Yuri Kim[2], Da Som Jeong[2,3], Yu Cheon Kim[2,3], Myoung Hee Kim[2,3,*], and Je-Yoel Cho[4,5,*]

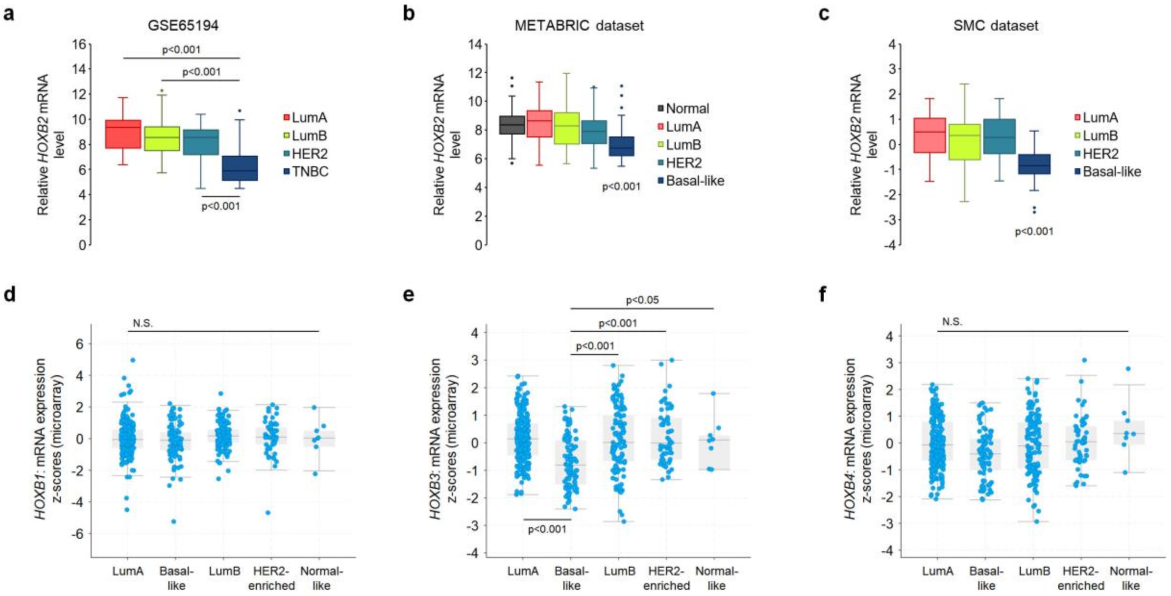

Figure S1. Expression levels of anterior *HOXB* cluster genes in different subtypes of breast cancer. **a-c** Analysis of public dataset GSE65194 (**a**), METABRIC dataset (**b**), and SMC dataset (**c**) for the expression of *HOXB2* in different subtypes of breast cancer. **d-f** Analysis of TCGA dataset (Nature 2012) for the expression of *HOXB1* (**d**), *HOXB3* (**e**), and *HOXB4* (**f**) in different breast cancer subtypes.

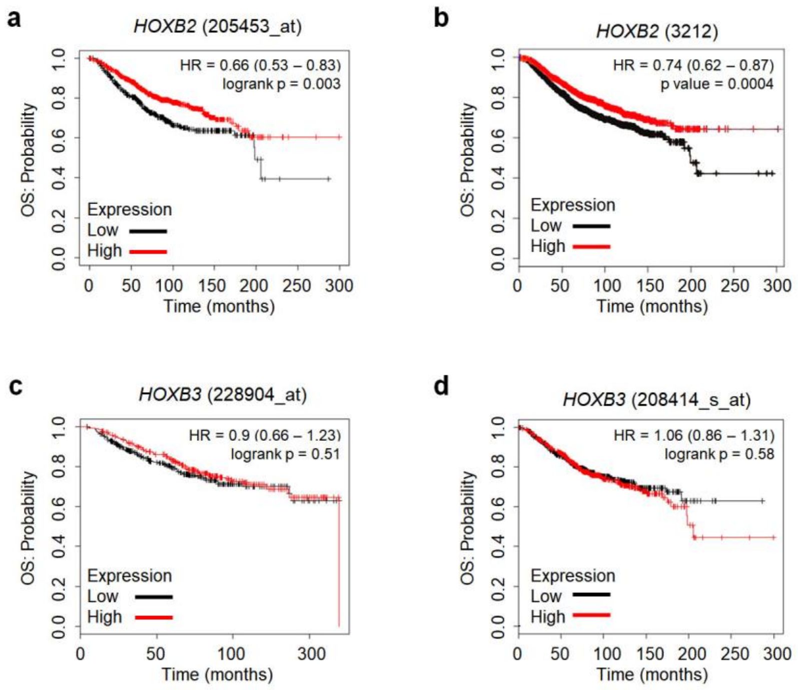

Figure S2. Kaplan-Meier overall survival (OS) analysis comparing high and low levels of *HOXB2* and *HOXB3* in breast cancer. **a, b** OS analyses of the relationship between survival time and *HOXB2* signature in breast cancer using (**a**) the Kaplan–Meier plotter and (**b**) Molecular Therapeutics for Cancer, Ireland (MTCI) online tools. For survival analysis, the *HOXB2* (205453_at for KM plot, 3212 for MTCI) probe was selected and analyzed. **c, d** OS analyses of the relationship between survival time and *HOXB3* signature in breast cancer using the Kaplan–Meier plotter online tool. For survival analysis, the *HOXB3* 228904_at (**c**) and 208414_at (**d**) probes were selected and analyzed.

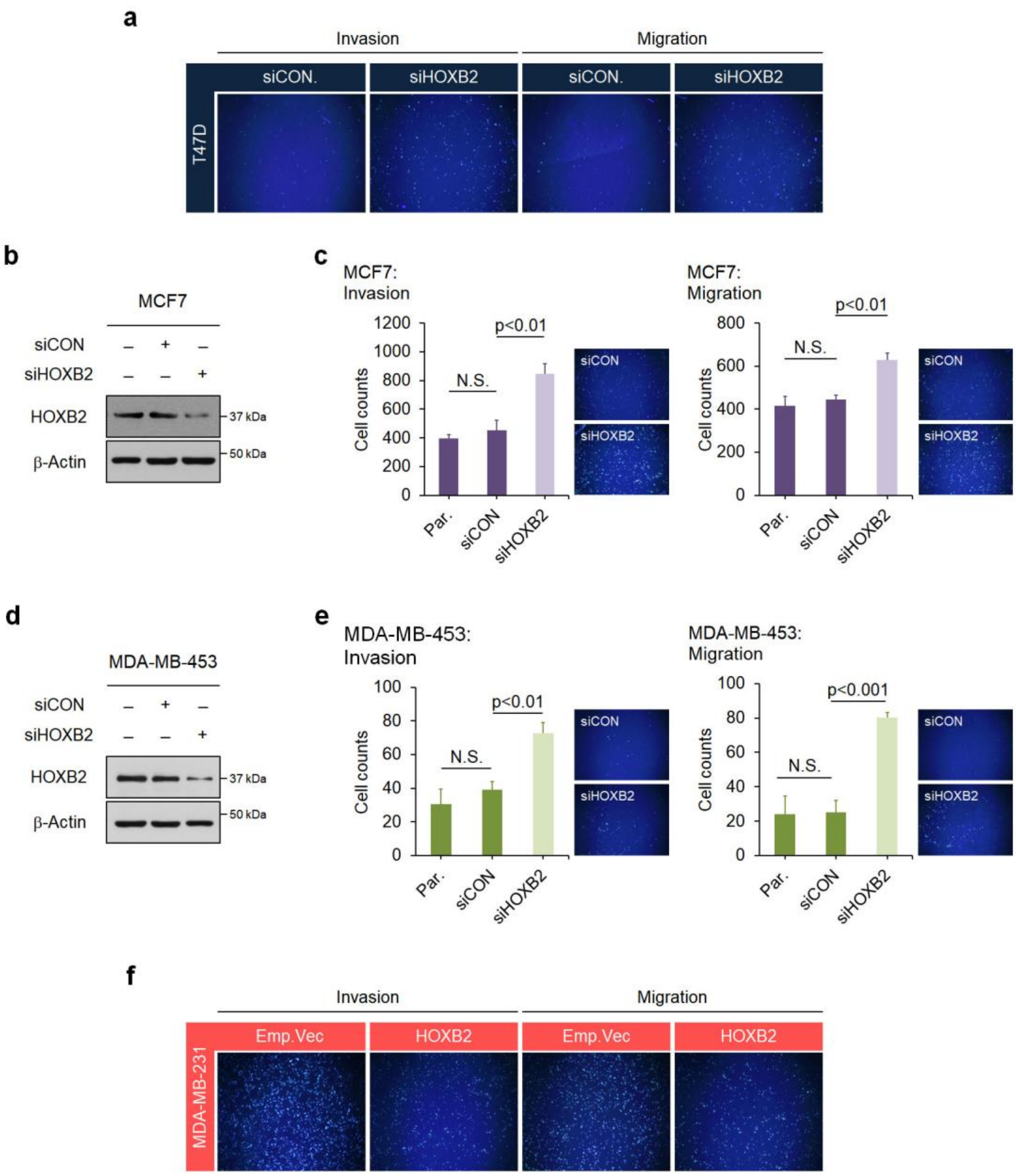

Figure S3. HOXB2 attenuates invasion and migration abilities in non-TNBC cells. **a** Transwell assays of T47D cells transfected with siCON or siHOXB2. The invaded and migrated cells were stained with DAPI. **b** Protein level of knocked-down HOXB2 in MCF7 breast cancer cells. **c** Transwell assays of MCF7 cells transfected with siCON or siHOXB2. The invaded (left panel) and migrated (right panel) cells were stained with DAPI and counted. **d** Protein level of knocked-down HOXB2 in MDA-MB-453 breast cancer cells. **e** Transwell

assays of MDA-MB-453 cells transfected with siCON or siHOXB2. The invaded (left panel) and migrated (right panel) cells were stained with DAPI and counted. **f** Transwell assays of MDA-MB-231 cells transfected with empty vector or HOXB2. The invaded and migrated cells were stained with DAPI.

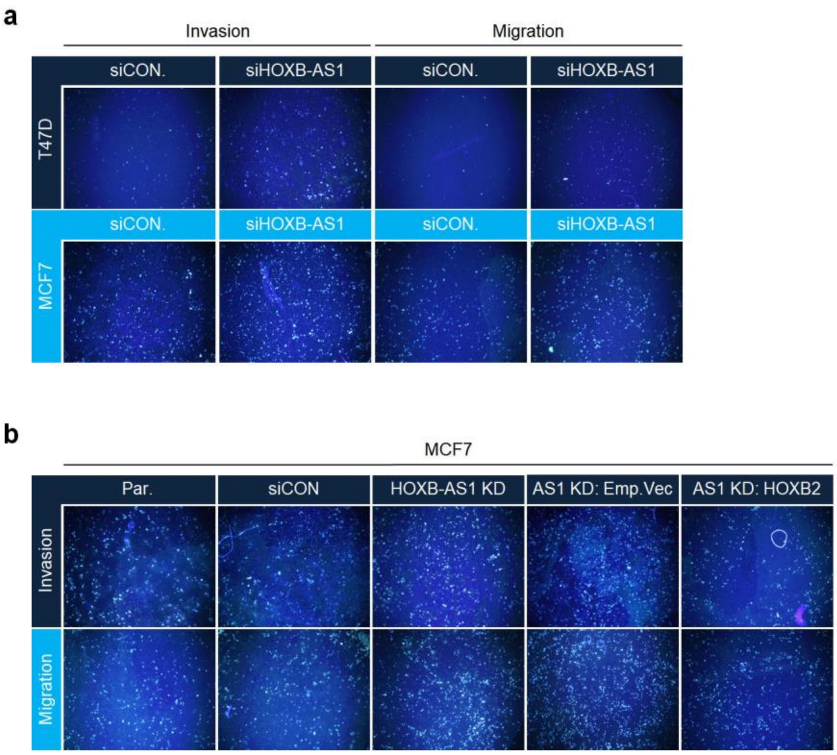

Figure S4. HOXB-AS1 attenuates invasion and migration abilities via mediating HOXB2 in non-TNBC cells. **a** Transwell assays of T47D/MCF7 cells transfected with siCON or siHOXB-AS1. The invaded and migrated cells were stained with DAPI. **b** Transwell assays of parent MCF7, MCF7:siCON, MCF7:siHOXB-AS1, MCF7:HOXB-AS1 KD:Emp.Vec, and MCF7:HOXB-AS1 KD:HOXB2 cells. The invaded and migrated cells were stained with DAPI.

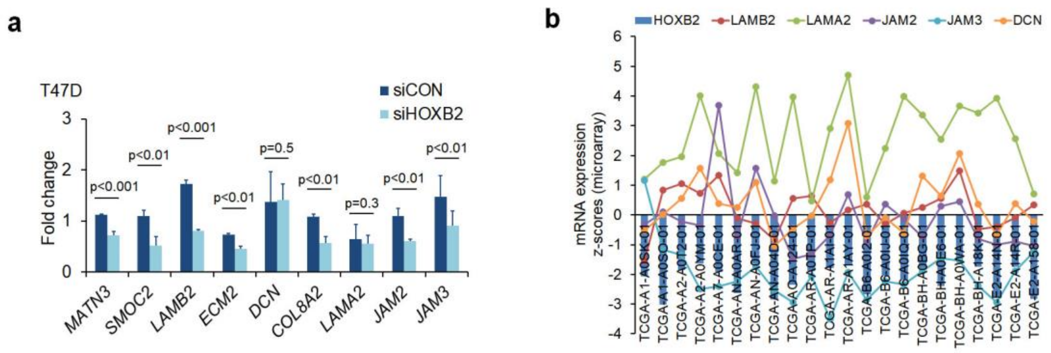

Figure S5. Expression levels of extracellular matrix (ECM) organization genes in HOXB2 dysregulated breast cancer. **a** Expression levels of nine extracellular matrix organization genes in T47D cells transfected with siCON or siHOXB2. **b** Expression levels of *HOXB2*, *LAMB2*, *LAMA2*, *JAM2*, *JAM3*, and *DCN* in patients with HOXB2-altered basal-like subtype breast cancer.

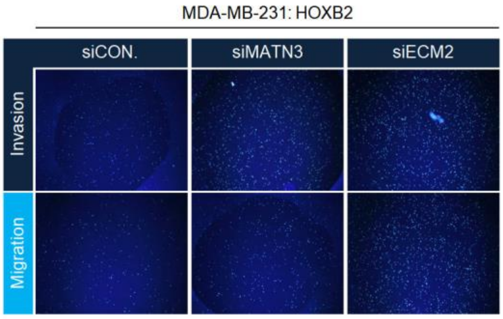

Figure S6. MATN3 and ECM2 are associated with aggressive phenotypes in TNBC. Transwell assays of MDA-MB-231:HOXB2 cells transfected with siCON or siMATN3, siECM2. The invaded and migrated cells were stained with DAPI.

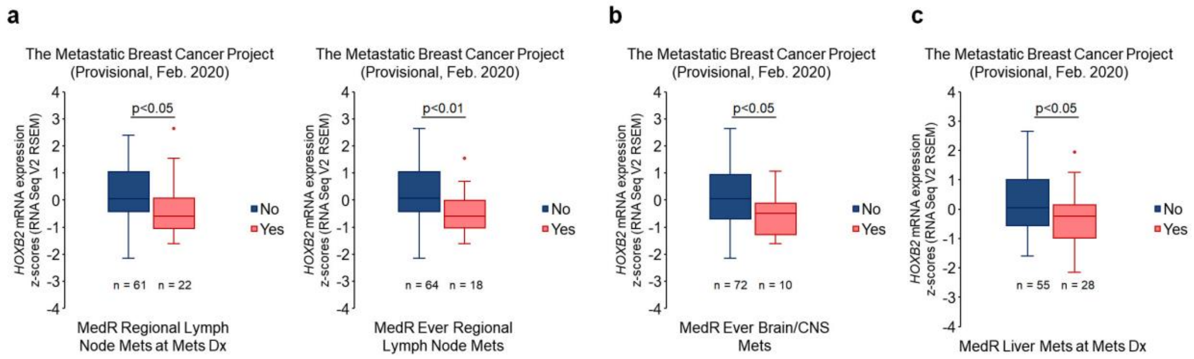

Figure S7. Overexpression of HOXB2 attenuates BC metastasis. **a** Analyses of The Metastatic Breast Cancer Project dataset (Provisional, Feb. 2020) for the expression of *HOXB2* in regional lymph node metastasis. Left panel: metastasis to regional lymph nodes at the time of the diagnosis of metastatic disease; Right panel: metastasis to regional lymph nodes noted during the patient's course of treatment. **b, c** Analyses of The Metastatic Breast Cancer Project dataset (Provisional, Feb. 2020) for the expression of *HOXB2* in brain/CNS (**b**) and liver (**c**) metastasis.

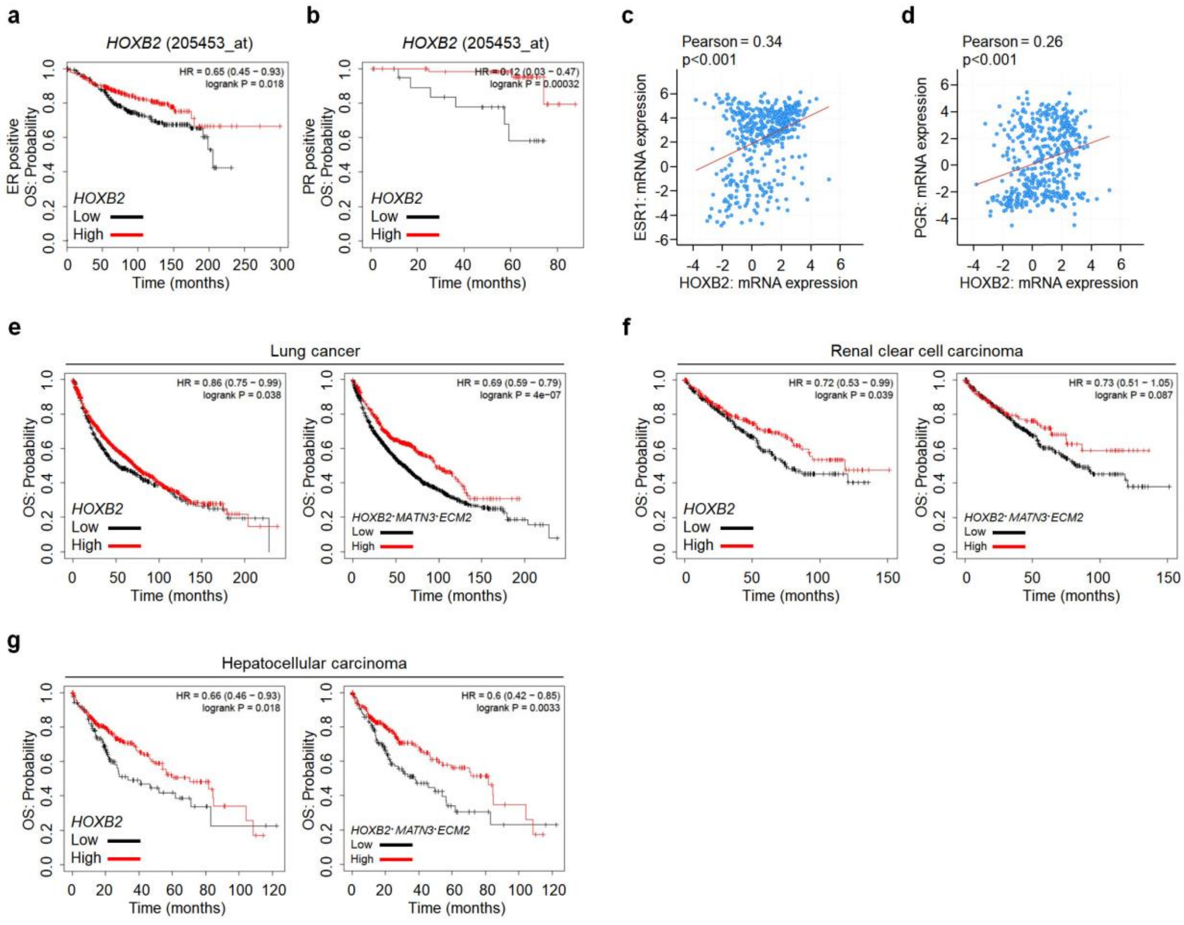

Figure S8. HOXB2, MATN3, and ECM2 are robust clinical markers for patients with various cancers. **a, b** Kaplan–Meier OS analyses comparing high and low levels of *HOXB2* in ER-positive (**a**), PR-positive (**b**) breast cancer. **c** Correlation curve between HOXB2 and ESR1 expression in the TCGA breast cancer tissue database. **d** Correlation curve between HOXB2 and PGR expression in the TCGA breast cancer tissue database. **e-g** Kaplan–Meier OS analyses comparing high and low levels of *HOXB2* or *HOXB2*/*MATN3*/*ECM2* expression in lung cancer (**e**), renal clear cell carcinoma (**f**), and hepatocellular carcinoma (**g**). For survival analysis, *HOXB2* (205453_at), *MATN3* (206091_at), and *ECM2* (206101_at) probes were selected and analyzed.

Table S1. Sequences of siRNAs.

	Sense (5'-3')	Antisense (5'-3')
siHOXB2 #1	CUGUCUUGGAGACAUUUCAUU	UGAAAUGUCUCCAAGACAGUU
siHOXB2 #2	CCUUGGAUGAAAGAGAAGAUU	UCUUCUCUUUCAUCCAAGGUU
siHOXB2 #3	GAAGAAAUCCGCCAAGAAAUU	UUUCUUGGCGGAUUUCUUCUU
siHOXB2 #4	GUCAAAGUCUGGUUUCAGAUU	UCUGAAACCAGACUUUGACUU
siHOXB2 #5	CCAGGAUUCACCUUUCCUUUU	AAGGAAAGGUGAAUCCUGGUU
siHOXB-AS1 #1	GCGAAAUUACAGGGAAUUCUU	GAAUUCCCUGUAAUUUCGCUU
siHOXB-AS1 #2	GGAAGAGGGAGUCGAAAGAUU	UCUUUCGACUCCCUCUUCCUU
siHOXB-AS1 #3	CGGAAGAGGAUUGUGAAGUUU	ACUUCACAAUCCUCUUCCGUU
siHOXB-AS1 #4	GUUUCGUGGAAGAAACCGAUU	UCGGUUUCUUCCACGAAACUU
siHOXB-AS1 #5	CGUGGAAGAAACCGAUAAUUU	AUUAUCGGUUUCUUCCACGUU
siMATN3 #1	CUGGAAUUCACCAAAGUGAUU	UCACUUUGGUGAAUUCCAGUU
siMATN3 #2	CUAAGGUGGCCAUCAUUGUUU	ACAAUGAUGGCCACCUUAGUU
siMATN3 #3	GAUAGGUGUGCUCUUAACAUU	UGUUAAGAGCACACCUAUCUU
siMATN3 #4	GACAGAAGUGGCUCUUAUCUU	GAUAAGAGCCACUUCUGUCUU
siMATN3 #5	GCUCGUAUCUUCAAAGACUUU	AGUCUUUGAAGAUACGAGCUU
siSMOC2 #1	CCUUCCUUUCCCGUUGUGAUU	UCACAACGGGAAAGGAAGGUU
siSMOC2 #2	CCGUUGUGAAUUUCAACGUUU	ACGUUGAAAUUCACAACGGUU
siSMOC2 #3	GCUAGAGAUUGCAUAUCGAUU	UCGAUAUGCAAUCUCUAGCUU
siSMOC2 #4	GAGGAAACUGCAAAGACGUUU	ACGUCUUUGCAGUUUCCUCUU
siSMOC2 #5	CCAAGAACGACAAUGUGGUUU	ACCACAUUGUCGUUCUUGGUU
siECM2 #1	GGCAUAACCAUGUACAACAUU	UGUUGUACAUGGUUAUGCCUU
siECM2 #2	CAAUCUUCAGGCUAUCGAUUU	AUCGAUAGCCUGAAGAUUGUU
siECM2 #3	CUCCUUUAGCCUGGAUAAAUU	UUUAUCCAGGCUAAAGGAGUU
siECM2 #4	CCUACAACAAGCUCUAUCAUU	UGAUAGAGCUUGUUGUAGGUU
siECM2 #5	CAAGAUACGCUGUGUUUCAUU	UGAAACACAGCGUAUCUUGUU
siCOL8A2 #1	CCAGUACCUGGAAAUGCCUUU	AGGCAUUUCCAGGUACUGGUU
siCOL8A2 #2	GCGUCUACUACUUUGCUUAUU	UAAGCAAAGUAGUAGACGCUU
siCOL8A2 #3	CCACCUAUACCUACGAUGAUU	UCAUCGUAGGUAUAGGUGGUU
siCOL8A2 #4	CCUAUACCUACGAUGAGUAUU	UACUCAUCGUAGGUAUAGGUU
siCOL8A2 #5	CCUACGAUGAGUACAAGAAUU	UUCUUGUACUCAUCGUAGGUU

Table S2. Primer sequences used for real-time qPCR and ChIP-PCR analyses.

	Real-time qPCR	
	Forward primer (5'-3')	Reverse primer (5'-3')
h-HOXB2	TTC ACC AGT ACG CTC TGT GC	AAA GAT AAC CGA GTG CCC AAT
h-CDH1	AGG ACC AGG ACT TTG ACT TG	ATT GCC CCA TTC GTT CAA GT
h-CDH2	CGT GAA GGT TTG CCA GTG TG	CGG ATT CCC ACA GGC TTG AT
h-ITGB4	TGA GCC ACT GGA GAG CC	TCA TGT CCG TCT GCG GG
h-VIM	CTG CCA ACC GGA ACA ATG AC	CAT TTC ACG CAT CTG GCG TT
h-MATN3	CTC TAT GCT GTG GGC GTG G	TGG CAC TGG TGT GTT CCA A
h-SMOC2	CAA AGC GGA CAC CAA GAA ACG	GTG TCT AGG AAC TGC CTT CGG
h-LAMB2	GTA CGT GGC AAC TGC TTC TG	GAA ATC CTG ACA CTG CTC GC
h-ECM2	CTC ACT GGC AAT TCC ATC GC	GGA CCT ATG CCT GAA GAA GTG A
h-DCN	GGG ATA GGC CCA GAA GTT CC	CAG AAC ACT GGA CCA CTC GAA
h-COL8A2	ACC CAA ATC TGC CCA AGT GA	AGA GGT TGA GCG AAG AAC CC
h-LAMA2	GAA CCC GCA GTG TCG AAT C	GGA CTC TGC CAC CAA GTG T
h-JAM2	ATT AGT GGC TCC AGC AGT TCC	CCA TGT GTA TTC AGG AGC TGG A
h-JAM3	CCA GTA GGC AAG ATG GCA AC	CCC AGA GTC GTC CTT GTG AA
h-β-Actin	CAT GTT TGA GAC CTT CAA CAC CCC	GCC ATC TCC TGC TCG AAG TCT AG
	ChIP-PCR	
	Forward primer (5'-3')	Reverse primer (5'-3')
HOXB2 #1	CCA CTA TCC CTG TTA CCC TCC	GGC TTG AGT GGG TAC ATG C
HOXB2 #2	CTA TTA AAC CCA GGA CTC CAG C	GCT GGC CAC GTA AAG AAG G
HOXB2 #3	CCT CTC TAG TCT ACA GCC CC	CCC GTA TCT GTG TAG GGG AA
MATN3 #1	GCG AGG CTC GGA TTT AAA GG	CAG ACA TTC CGT TTC TGC CG
MATN3 #2	TCA GGA AGG GTT TGC TCA CT	GAT GTG GGT CTC CTG AAG CTC
ECM2 #1	AGG CTT TGC TTG GTG TCA TTT	TAT CCC CAA GGG AGG CAG TT
ECM2 #2	TCA GCC AAG AAA GTA ACC AGG G	TCC AAC TCT GCC TTT TCA GC
Gene desert	TGG TGG TCT GCC TTC TGC CAG T	TCA CGT GGG AGG AAG AAG TAG GGC
